# Exact expressions and numerical evaluation of average evolvability measures for characterizing and comparing $\textbf{G}$ matrices

**DOI:** 10.1007/s00285-023-01930-8

**Published:** 2023-05-22

**Authors:** Junya Watanabe

**Affiliations:** Department of Earth Sciences, University of Cambridge, Downing Street, Cambridge, CB2 3EQ UK

**Keywords:** Evolutionary constraint, Phenotypic integration, Quadratic form, Quantitative genetics, Random skewers, Zonal polynomial, 92D15, 62P10, 92-08

## Abstract

Theory predicts that the additive genetic covariance ($\textbf{G}$) matrix determines a population’s short-term (in)ability to respond to directional selection—evolvability in the Hansen–Houle sense—which is typically quantified and compared via certain scalar indices called evolvability measures. Often, interest is in obtaining the averages of these measures across all possible selection gradients, but explicit formulae for most of these average measures have not been known. Previous authors relied either on approximations by the delta method, whose accuracy is generally unknown, or Monte Carlo evaluations (including the random skewers analysis), which necessarily involve random fluctuations. This study presents new, exact expressions for the average conditional evolvability, average autonomy, average respondability, average flexibility, average response difference, and average response correlation, utilizing their mathematical structures as ratios of quadratic forms. The new expressions are infinite series involving top-order zonal and invariant polynomials of matrix arguments, and can be numerically evaluated as their partial sums with, for some measures, known error bounds. Whenever these partial sums numerically converge within reasonable computational time and memory, they will replace the previous approximate methods. In addition, new expressions are derived for the average measures under a general normal distribution for the selection gradient, extending the applicability of these measures into a substantially broader class of selection regimes.

**Supplementary Information:**

The online version contains supplementary material available at 10.1007/s00285-023-01930-8.

## Introduction

The quantitative genetic theory of multivariate trait evolution provides a powerful framework to analyze and predict phenotypic evolution (Steppan et al. 2002; Blows 2007; Blows and Walsh 2009; Walsh and Blows 2009; Teplitsky et al. 2014). At the core of the theory is the Lande equation, which describes a population’s response to directional selection under certain simplifying conditions (Lande 1979; Lande and Arnold 1983):

$$\begin{aligned} \Delta \bar{\textbf{z}}= \textbf{G} \varvec{\upbeta }, \end{aligned}$$

where $\varvec{\upbeta }$ is a *p*-dimensional selection gradient vector (partial regression coefficients of trait values to relative fitness), $\textbf{G}$ is a $p \times p$ (additive) genetic covariance matrix, and $\Delta \bar{\textbf{z}}$ is a *p*-dimensional vector of per-generation change of the population’s mean trait values. (Throughout the paper, *p* denotes the (nominal) number of the traits/variables analyzed, and all vectors are column vectors without transposition.) In light of this theory, the $\textbf{G}$ matrix is supposed to represent the population’s (in)ability to respond to directional selection—what has been termed evolvability by Hansen, Houle and colleagues (Houle 1992; Hansen 2003; Hansen et al. 2003a, b, 2011; Hansen and Houle 2008; Hansen and Pélabon 2021).^1^ The theory has motivated extensive investigations into aspects of the $\textbf{G}$ matrix or its substitutes, ranging from theoretical and simulation-based analyses (e.g., Pavlicev and Hansen 2011; Pavlicev et al. 2011; Jones et al. 2012; Chevin 2013; Melo and Marroig 2015; Hansen et al. 2019) to empirical inter-population/specific comparisons of trait covariation and its expansion to inter-population divergences (e.g., Cheverud 1982, 1989; Schluter 1996; Porto et al. 2009; Marroig et al. 2009; Rolian 2009; Bolstad et al. 2014; Haber 2016; Puentes et al. 2016; Costa e Silva et al. 2020; Machado 2020; McGlothlin et al. 2022; Opedal et al. 2022, 2023; Hubbe et al. 2023).

**Fig. 1:**
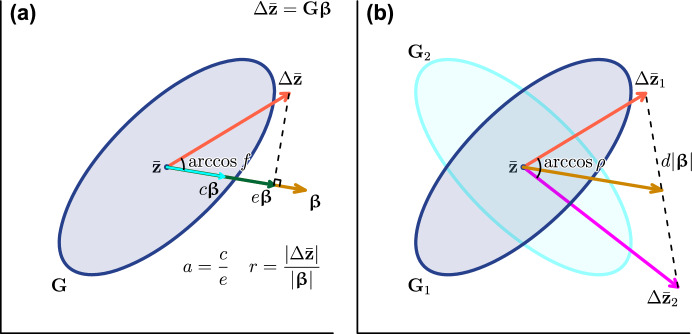
Geometric interpretations of evolvability measures. In a hypothetical bivariate trait space, genetic covariance matrices $\textbf{G}$ are schematically represented by equiprobability ellipses around the mean phenotype $\bar{\textbf{z}}$, and selection gradients $\varvec{\upbeta }$ by acute-headed brown arrows. Response vectors $\Delta \bar{\textbf{z}}$ are matrix products of $\textbf{G}$ and $\varvec{\upbeta }$, represented by broad-headed red/magenta arrows. (**a**) One-matrix measures: evolvability *e* is the norm of $\Delta \bar{\textbf{z}}$ projected on $\varvec{\upbeta }$, standardized by the norm of the latter, $|\varvec{\upbeta } |$; conditional evolvability *c* is the same but when $\Delta \bar{\textbf{z}}$ is constrained along $\varvec{\upbeta }$; autonomy *a* is the ratio of *c* to *e*; respondability *r* is $|\Delta \bar{\textbf{z}} |$ standardized by $|\varvec{\upbeta } |$; and flexibility *f* is the similarity in direction (cosine of the angle) between $\Delta \bar{\textbf{z}}$ and $\varvec{\upbeta }$. (**b**) Two-matrix comparison measures: response difference *d* is the distance between the end points of the two $\Delta \bar{\textbf{z}}$’s standardized by $|\varvec{\upbeta } |$, and response correlation $\rho$ is their similarity in direction

Based on this framework, and following earlier theoretical developments (Hansen 2003; Hansen et al. 2003a, b), Hansen and Houle (2008) proposed several scalar indices to capture certain aspects of the multivariate evolvability represented in $\textbf{G}$ (see also Kirkpatrick 2009; Marroig et al. 2009). Of these, (unconditional) evolvability *e*, conditional evolvability *c*, autonomy *a*, integration *i*, respondability *r*, and flexibility *f* (due to Marroig et al. 2009) concern a single $\textbf{G}$ matrix (Fig. 1A), whereas response difference *d* concerns comparison between two $\textbf{G}$ matrices. Related to the latter is the use of response correlation $\rho$ for matrix comparison (Cheverud 1996; Cheverud and Marroig 2007) (Fig. 1B). Notably, all these indices are simple or multiple ratios of quadratic forms in $\varvec{\upbeta }$ (see below). In this paper, they are collectively called evolvability measures. Many of them can be related to the rate of adaptation or evolutionary constraint/bias (e.g., Hansen and Houle 2008; Chevin 2013; Bolstad et al. 2014; Hansen et al. 2019), providing means to characterize and compare $\textbf{G}$ matrices in biologically meaningful ways.

The evolvability measures are primarily defined with respect to a given selection gradient $\varvec{\upbeta }$. In this sense, they represent aspects of $\textbf{G}$ matrices under a fixed, deterministic selection. In many theoretical and comparative analyses of evolvability (see references above), however, interest is often in characterizing $\textbf{G}$ matrices without referring to $\varvec{\upbeta }$. For this purpose, it is sensible to take the expectation of an evolvability measure assuming a certain probability distribution of $\varvec{\upbeta }$. Most typically, although not necessarily, the uniform distribution on a unit hypersphere is assumed for this purpose, representing a completely randomly directed and uncorrelated selection regime. The resultant quantities are called average measures hereafter.^2^ Biologically speaking, the average measures may be regarded as representations of general evolvability (in the sense of Riederer et al. 2022), as compared to specific evolvability which is represented by the evolvability measures as functions of a fixed $\varvec{\upbeta }$.

However, there is a major obstacle to using average measures for practical or even theoretical investigations. That is, most of the average measures lack known explicit, exact expressions beyond as expectations, except for the average evolvability ${\bar{e}}$ and, under certain special conditions, the average conditional evolvability ${\bar{c}}$ and the average respondability ${\bar{r}}$ (Hansen and Houle 2008; Kirkpatrick 2009). Consequently, previous studies had to rely on approximations of average measures. Presumably the most widely used approximation method is Monte Carlo evaluation (e.g., Marroig et al. 2009; Bolstad et al. 2014; Haber 2016; Grabowski and Porto 2017), in which a large number of $\varvec{\upbeta }$ is randomly generated on a computer and the resultant values calculated with the primary definition are literally averaged to yield an estimate of average measure. This has long been done in the so-called random skewers analysis for matrix comparison using response correlation $\rho$ (e.g., Cheverud 1996; Cheverud and Marroig 2007; Revell 2007; see also Rohlf 2017). It is implemented in the R packages evolvability (Bolstad et al. 2014) and evolqg (Melo et al. 2016) for calculating various average measures. This method necessarily involves random fluctuations in the estimate and can take a large computational time, although the latter is becoming less of a concern with modern-day computers. On the other hand, Hansen and Houle (2008, 2009) themselves provided approximate expressions for average measures based on the delta method (which they called “analytical” or “standard” approximations). Apart from ill-defined notations used therein, a practical problem there is that the accuracy of this sort of approximation is generally unknown, so a separate Monte Carlo evaluation is usually required to ascertain whether the delta method approximation has an acceptable accuracy. This approximation has been used in some subsequent studies (e.g., Hansen and Voje 2011; Brommer 2014; Delahaie et al. 2017; Saltzberg et al. 2022) and is available in the evolvability package. Apart from these methods, Kirkpatrick (2009) used numerical integration to evaluate his average selection response, a measure equivalent to respondability (below), but this method has not been widely used in evaluating average measures.

This technical paper provides exact expressions for the following average measures: average evolvability ${\bar{e}}$, average conditional evolvability ${\bar{c}}$, average autonomy ${\bar{a}}$, average respondability ${\bar{r}}$, average flexibility ${\bar{f}}$, average response difference ${\bar{d}}$, and average response correlation ${\bar{\rho }}$. These expressions are derived from existing and new results on the moments of simple or multiple ratios of quadratic forms in normal variables. They are expressed as infinite series involving zonal or invariant polynomials of matrix arguments, and in most cases can be numerically evaluated as their partial sums to yield quasi-exact values. For some of the expressions, an upper bound for the truncation error is available. In addition to the special condition where $\varvec{\upbeta }$ is spherically distributed as assumed in most previous studies (but see also Chevin 2013), this study also concerns the general condition that $\varvec{\upbeta }$ is normally distributed with potentially nonzero mean and nonspherical covariance. The latter condition can be of substantial biological interest, as it can model a fairly wide range of random directional and/or correlated selection regimes.

The paper is structured as follows. Section 2 first reviews the definition of the evolvability measures; some of them are redefined to accommodate potentially singular $\textbf{G}$ matrices. After reviewing some known results on the moment of a ratio of quadratic forms, it then provides new expressions for average evolvability measures under the spherical distribution of $\varvec{\upbeta }$. Section 3 presents a new R implementation of the analytic results and evaluate its performance by numerical experiments. Section 4 concludes the main body of the paper by adding some theoretical and practical considerations on the use of average measures. As the zonal and invariant polynomials appear to have rarely been used in the biological literature, Appendix A gives a brief overview on their theories, providing a basis for the present mathematical results. Appendix B states a new result on the moment of a multiple ratio of quadratic forms in normal variables, and Appendix C presents new expressions for average measures under the general normal distribution of $\varvec{\upbeta }$. Appendix D clarifies connections to previous results derived under special conditions (Hansen and Houle 2008; Kirkpatrick 2009).

## Theory

### Notations

In the following discussion, it is convenient to define the linear combination of the variables (traits) along the direction of $\varvec{\upbeta }$. For this purpose, consider the decomposition $\varvec{\upbeta } = |\varvec{\upbeta } | \textbf{u}$, where $|\cdot |$ is the vector norm or length, $|\textbf{a} | = \sqrt{\textbf{a}^T \textbf{a}}$ for an arbitrary vector $\textbf{a}$ (the superscript ${}^T$ denotes matrix transpose), and $\textbf{u}$ is a unit vector: $\textbf{u}^T \textbf{u} = 1$. Define the $p \times (p - 1)$ matrix $\textbf{U}_{(-1)}$ so that the matrix $\textbf{U} = (\textbf{u}, \textbf{U}_{(-1)})$ is orthogonal. With these, the orthogonal linear transform of the variables $\textbf{z}^* = \textbf{U}^T \textbf{z}$ will be considered; the first entry of this new vector $\textbf{u}^T \textbf{z}$ represents the score along the direction of $\varvec{\upbeta }$. Note that the covariance matrix of the new variables $\textbf{z}^*$ can be written as

$$\begin{aligned} \textbf{G}^* = \textbf{U}^T \textbf{G} \textbf{U} = \begin{pmatrix} \textbf{u}^T \textbf{G} \textbf{u} &{} \textbf{u}^T \textbf{G} \textbf{U}_{(-1)} \\ \textbf{U}_{(-1)}^T \textbf{G} \textbf{u} &{} \textbf{U}_{(-1)}^T \textbf{G} \textbf{U}_{(-1)} \end{pmatrix}. \end{aligned}$$

Previous authors (Hansen and Houle 2008; Marroig et al. 2009; Grabowski and Porto 2017) defined some evolvability measures under the (sometimes implicit) constraint $|\varvec{\upbeta } | = 1$. To avoid potential confusion, this standardized vector is always denoted by $\textbf{u}$ herein. Throughout this paper, $\textbf{G}$ is assumed to be given rather than random and validly constructed as a covariance matrix, that is, symmetric and nonnegative definite (i.e., either positive definite or positive semidefinite, in the terminology of Schott 2016).

Although Hansen and Houle (2008) assumed $\textbf{G}$ to be positive definite, this assumption is not imposed here to accommodate potentially singular (and positive semidefinite) $\textbf{G}$ where possible. This is done by using the generalized inverse; for a matrix $\textbf{A}$, a generalized inverse $\textbf{A}^{-}$ is such a matrix that satisfies $\textbf{A} \textbf{A}^{-} \textbf{A} = \textbf{A}$. If $\textbf{A}$ is nonsingular, $\textbf{A}^{-} = \textbf{A}^{-1}$.

For notational simplicity, the range or column space and the null space of a nonzero $p \times q$ matrix $\textbf{A}$ are defined: $R \left( \textbf{A} \right) = \{ \textbf{y}: \textbf{y} = \textbf{A} \textbf{x}, \textbf{x} \in {\mathbb {R}}^q \}$, and $N \left( \textbf{A} \right) = \{ \textbf{x}: \textbf{0}_p = \textbf{A} \textbf{x}, \textbf{x} \in {\mathbb {R}}^q \}$, where $\textbf{0}_p$ is the *p*-dimensional vector of zeros. When $\textbf{A}$ is nonnegative definite, these are the spaces spanned by the eigenvectors corresponding to its nonzero and zero eigenvalues, respectively. The *p*-dimensional identity matrix is denoted by $\textbf{I}_p$.

### Evolvability measures: fixed selection

Evolvability $e(\varvec{\upbeta })$ is the variance in $\textbf{G}$ along the direction of selection $\textbf{u}$, or equivalently the norm of the response vector $\Delta \bar{\textbf{z}}$ projected onto $\varvec{\upbeta }$, standardized by $|\varvec{\upbeta } |$ (Hansen and Houle 2008) (Fig. 1):

1 $$\begin{aligned} e(\varvec{\upbeta })&= \frac{ \varvec{\upbeta }^T \Delta \bar{\textbf{z}}}{ \varvec{\upbeta }^T \varvec{\upbeta } } \nonumber \\&= \frac{ \varvec{\upbeta }^T \textbf{G} \varvec{\upbeta } }{ \varvec{\upbeta }^T \varvec{\upbeta } } \end{aligned}$$

2 $$\begin{aligned}&= \textbf{u}^T \textbf{G} \textbf{u}. \end{aligned}$$

The numerator in (1) can be related to the rate of adaptation (change in mean fitness) under simple directional selection (Agrawal and Stinchcombe 2009; Chevin 2013).

Conditional evolvability $c(\varvec{\upbeta })$ is conceptually defined as the variance along $\textbf{u}$ when evolution in other traits/directions is not allowed due to stabilizing selection (Hansen 2003; Hansen et al. 2003a, 2019). With above notations, it is the conditional variance of the first element of $\textbf{z}^*$ given the other elements (redefined here after Hansen and Houle 2008):

3 $$\begin{aligned} c(\varvec{\upbeta })&= \textbf{u}^T \textbf{G} \textbf{u} - \textbf{u}^T \textbf{G} \textbf{U}_{(-1)} \left( \textbf{U}_{(-1)}^T \textbf{G} \textbf{U}_{(-1)} \right) ^{-} \textbf{U}_{(-1)}^T \textbf{G} \textbf{u} \nonumber \\&= (\textbf{u}^T \textbf{G}^{-} \textbf{u})^{-} \quad \text {if } \varvec{\upbeta }, \textbf{u} \in R \left( \textbf{G} \right) , \end{aligned}$$

4 $$\begin{aligned}&= \frac{ \varvec{\upbeta }^T \varvec{\upbeta } }{ \varvec{\upbeta }^T \textbf{G}^{-} \varvec{\upbeta } }. \end{aligned}$$

Here, the equation of (3) is from well-known results on the (generalized) inverse of a partitioned matrix (e.g., Schott 2016, theorems 7.1 and 7.12), for which Hansen and Houle (2008) restated a proof in nonsingular $\textbf{G}$. A lack of conditional variance along $\varvec{\upbeta }$ results in $c(\varvec{\upbeta }) = 0$, which happens when $\textbf{G}$ is singular and $\varvec{\upbeta } \notin R \left( \textbf{G} \right)$; the expressions (3) and (4) do not hold in this case.

Autonomy $a(\varvec{\upbeta })$ is defined as the complement of the squared multiple correlation of the first element of $\textbf{z}^*$ with respect to the other elements:

5 $$\begin{aligned} a(\varvec{\upbeta })&= 1 - (\textbf{u}^T \textbf{G} \textbf{u})^{-1} \textbf{u}^T \textbf{G} \textbf{U}_{(-1)} \left( \textbf{U}_{(-1)}^T \textbf{G} \textbf{U}_{(-1)} \right) ^{-} \textbf{U}_{(-1)}^T \textbf{G} \textbf{u} = \frac{c(\varvec{\upbeta })}{e(\varvec{\upbeta })} \nonumber \\&= (\textbf{u}^T \textbf{G} \textbf{u} \textbf{u}^T \textbf{G}^{-} \textbf{u})^{-1} \quad \text {if } c(\varvec{\upbeta }) \ne 0, \end{aligned}$$

6 $$\begin{aligned}&= \frac{(\varvec{\upbeta }^T \varvec{\upbeta })^2}{\varvec{\upbeta }^T \textbf{G} \varvec{\upbeta } \varvec{\upbeta }^T \textbf{G}^{-} \varvec{\upbeta }}. \end{aligned}$$

This quantity is undefined when $e(\varvec{\upbeta }) = \textbf{u}^T \textbf{G} \textbf{u} = 0$. Hansen and Houle (2008) also defined the integration $i(\varvec{\upbeta })$, which is the squared correlation just mentioned: $i(\varvec{\upbeta }) = 1 - a(\varvec{\upbeta })$.

Respondability $r(\varvec{\upbeta })$ is the magnitude of response standardized by that of selection, or the ratio of the norms of $\Delta \bar{\textbf{z}}$ and $\varvec{\upbeta }$:

7 $$\begin{aligned} r(\varvec{\upbeta })&= \frac{|\Delta \bar{\textbf{z}} |}{|\varvec{\upbeta } |} \nonumber \\&= \sqrt{ \frac{\varvec{\upbeta }^T \textbf{G}^2 \varvec{\upbeta }}{\varvec{\upbeta }^T \varvec{\upbeta }} } . \end{aligned}$$

The definition and terminology of *r* here follows Hansen and Houle (2008), but it should be noted that Kirkpatrick (2009) independently devised an equivalent measure as the (relative) selection response (*R* in the latter author’s notation) with a slightly different standardization.

Flexibility $f(\varvec{\upbeta })$ quantifies similarity in direction between $\Delta \bar{\textbf{z}}$ and $\varvec{\upbeta }$ in terms of vector correlation (cosine of the angle formed by two vectors), and is also the ratio of $e(\varvec{\upbeta })$ to $r(\varvec{\upbeta })$ (Hansen and Houle 2008; Marroig et al. 2009):

8 $$\begin{aligned} f(\varvec{\upbeta })&= \frac{e(\varvec{\upbeta })}{r(\varvec{\upbeta })} = \frac{ \varvec{\upbeta }^T \Delta \bar{\textbf{z}}}{ |\varvec{\upbeta } | |\Delta \bar{\textbf{z}} | } \nonumber \\&= \frac{ \varvec{\upbeta }^T \textbf{G} \varvec{\upbeta } }{ \sqrt{ \varvec{\upbeta }^T \varvec{\upbeta } \varvec{\upbeta }^T \textbf{G}^2 \varvec{\upbeta } } }. \end{aligned}$$

This quantity is undefined if $|\Delta \bar{\textbf{z}} | = 0$, i.e., $\varvec{\upbeta } \in N \left( \textbf{G} \right)$. Otherwise, $0 < f(\varvec{\upbeta }) \le 1$ from the nonnegative definiteness of $\textbf{G}$ and the definition of vector correlation. The term flexibility was coined by Marroig et al. (2009) due to Hansen’s suggestion, although the use of the vector correlation or angle was suggested in a few different works (Blows and Walsh 2009; Rolian 2009).

All the above indices are one-matrix measures that primarily concern characterization of a single $\textbf{G}$ matrix. On the other hand, the following two indices concern pairwise comparisons between two $\textbf{G}$ matrices in terms of (dis)similarity of the response vectors given the same $\varvec{\upbeta }$. The two $\textbf{G}$ matrices and $\Delta \bar{\textbf{z}}$ are denoted here with subscripts: $\textbf{G}_i$, $\Delta \bar{\textbf{z}}_i$. Response difference $d(\varvec{\upbeta })$ is a measure of dissimilarity, defined as a standardized difference between the two response vectors (Hansen and Houle 2008):

9 $$\begin{aligned} d(\varvec{\upbeta })&= \frac{| \Delta \bar{\textbf{z}}_1 - \Delta \bar{\textbf{z}}_2 |}{|\varvec{\upbeta } |} \nonumber \\&= \sqrt{ \frac{\varvec{\upbeta }^T \left( \textbf{G}_1 - \textbf{G}_2 \right) ^2 \varvec{\upbeta }}{\varvec{\upbeta }^T \varvec{\upbeta }}}. \end{aligned}$$

Response correlation $\rho (\varvec{\upbeta })$ is a measure of similarity in direction, defined as the vector correlation between the two response vectors (e.g., Cheverud 1996; Cheverud and Marroig 2007):

10 $$\begin{aligned} \rho (\varvec{\upbeta })&= \frac{ \Delta \bar{\textbf{z}}_1^T \Delta \bar{\textbf{z}}_2 }{ |\Delta \bar{\textbf{z}}_1 | |\Delta \bar{\textbf{z}}_2 | } \nonumber \\&= \frac{ \varvec{\upbeta }^T \textbf{G}_1 \textbf{G}_2 \varvec{\upbeta } }{ \sqrt{ \varvec{\upbeta }^T \textbf{G}_1^2 \varvec{\upbeta } \varvec{\upbeta }^T \textbf{G}_2^2 \varvec{\upbeta }}}. \end{aligned}$$

This quantity is undefined when at least one of $|\Delta \bar{\textbf{z}}_1 |$ and $|\Delta \bar{\textbf{z}}_2 |$ is 0. Otherwise, $-1 \le \rho (\varvec{\upbeta }) \le 1$.^3^

### Ratio of quadratic forms

A key to derive expressions for average measures is that the measures defined above are simple or multiple ratios of quadratic forms in $\varvec{\upbeta }$ or $\textbf{u}$. Ratios of quadratic forms, especially those in normal random variables, play a pivotal role in statistics, as many practically important test statistics can be written as a ratio of quadratic forms. Naturally, there is a vast body of literature regarding the distribution and moments of quadratic forms and ratios thereof (e.g., Magnus 1986; Jones 1986, 1987; Smith 1989, 1993; Mathai and Provost 1992; Hillier 2001; Forchini 2002; Hillier et al. 2009, 2014; Bao and Kan 2013).

Regarding the ratio of quadratic forms in normal variables, there are several equivalent ways to derive its moments. One starts from the joint moment-generation function (e.g., Cressie et al. 1981; Meng 2005) of quadratic forms, and typically results in integrations which does not have simple closed-form expressions (e.g., Magnus 1986; Jones 1986, 1987; Gupta and Kabe 1998). Another way is to expand a ratio into infinite series, which in turn is integrated using the zonal and invariant polynomials (e.g., Smith 1989, 1993; Hillier 2001; Hillier et al. 2009, 2014). (See Bao and Kan (2013) for connections between these two approaches.) The latter way typically yields an infinite series including zonal or invariant polynomials, which can be evaluated with reasonable speed and accuracy with the aid of recent algorithmic developments (Hillier et al. 2009, 2014; Bao and Kan 2013). This is the way followed in the present paper. The zonal polynomials are a special form of homogeneous polynomials in eigenvalues of a matrix which generalize powers of scalars into symmetric matrices (e.g., James 1960, 1964; Muirhead 1982, 2006; Gross and Richards 1987; Mathai et al. 1995). The invariant polynomials are a generalization of the zonal polynomials into multiple matrix arguments (Davis 1979, 1980; Chikuse 1980, 2003; Chikuse and Davis 1986b). A brief overview on these mathematical tools is provided in Appendix A.

A general result for the moments of a simple ratio of quadratic forms in normal variables is restated in the following proposition.

#### Proposition 1

(Smith 1989; Bao and Kan 2013) Let $\textbf{x} \sim N_p \left( \varvec{\upmu }, \textbf{I}_p \right)$, $\textbf{A}$ be a $p \times p$ symmetric matrix, $\textbf{B}$ be a $p \times p$ nonnegative definite matrix, and *m*, *n* be positive real numbers. When the expectation of $\frac{(\textbf{x}^T \textbf{A} \textbf{x})^m}{(\textbf{x}^T \textbf{B} \textbf{x})^n}$ exists, it can be written as

11 $$\begin{aligned} {{\,\textrm{E}\,}}\! \left( \frac{(\textbf{x}^T \textbf{A} \textbf{x})^m}{(\textbf{x}^T \textbf{B} \textbf{x})^n} \right)&= 2^{m - n} \alpha _{\textbf{A}}^{-m} \alpha _{\textbf{B}}^{n} e^{ -\frac{ \varvec{\upmu }^T \varvec{\upmu } }{2} } \sum _{i=0}^{\infty } \sum _{j=0}^{\infty } \sum _{k=0}^{\infty } \frac{ \left( -m \right) _{i} \left( n \right) _{j} \Gamma \left( \frac{p}{2} + m - n + k \right) }{ 2^k \left( \frac{1}{2} \right) _{k} \Gamma \left( \frac{p}{2} + i + j + k \right) } \nonumber \\&\qquad \cdot \frac{\left( \frac{1}{2} \right) _{i+j+k} }{ i! j! k! } C^{\left[ {i} \right] , \left[ {j} \right] , \left[ {k} \right] }_{\left[ {i+j+k} \right] } \! \left( \textbf{I}_p - \alpha _{\textbf{A}} \textbf{A} , \textbf{I}_p - \alpha _{\textbf{B}} \textbf{B} , \varvec{\upmu } \varvec{\upmu }^T \right) , \end{aligned}$$

where $\alpha _{\textbf{A}}$ and $\alpha _{\textbf{B}}$ are any positive constants that satisfy $0< \alpha _{\textbf{A}} < 2 / \lambda _{\max } \! \left( \textbf{A} \right)$ and $0< \alpha _{\textbf{B}} < 2 / \lambda _{\max } \! \left( \textbf{B} \right)$, with $\lambda _{\max } \! \left( \cdot \right)$ denoting the largest eigenvalue of the argument matrix, $\left( a \right) _{k} = a (a + 1) \dots (a + k - 1)$, and $C^{\left[ {i} \right] , \left[ {j} \right] , \left[ {k} \right] }_{\left[ {i+j+k} \right] } \! \left( \cdot , \cdot , \cdot \right)$ are the (*i*, *j*, *k*)-th top-order invariant polynomials (whose explicit form is presented in Appendix A).

#### Remark

Conditions for the existence of the moment are stated in Bao and Kan (2013, proposition 1 therein): when $\textbf{B}$ is positive definite, the moment exists if and only if $\frac{p}{2} + m > n$; and when $\textbf{B}$ is positive semidefinite, slightly different conditions apply, depending on the rank of $\textbf{B}$ and structures of $\textbf{A}$ and $\textbf{B}$. When *m* is an integer, the expressions can be simplified so that the summation for *i* disappears. Also, when $\varvec{\upmu } = \textbf{0}_p$, they substantially simplify as all terms with $k > 0$ are zero. If $\textbf{A}$ or $\textbf{B}$ is asymmetric, it can be symmetrized by using $\textbf{x}^T \textbf{A} \textbf{x} = \textbf{x}^T \textbf{A}^T \textbf{x} = \textbf{x}^T (\textbf{A} + \textbf{A}^T) \textbf{x} / 2$.

In practice, the above series can be approximated by its partial sum, with recursive algorithms to calculate $d_{i, j, k} \! \left( \textbf{A}_1 , \textbf{A}_2 , \textbf{A}_3 \right) = \frac{ \left( \frac{1}{2} \right) _{i + j + k} }{ i! j! k!} C^{\left[ {i} \right] , \left[ {j} \right] , \left[ {k} \right] }_{\left[ {i + j + k} \right] } \! \left( \textbf{A}_1 , \textbf{A}_2 , \textbf{A}_3 \right)$ (Chikuse 1987; Hillier et al. 2009, 2014; Bao and Kan 2013). General covariance structures for $\textbf{x}$ can be accommodated under certain conditions by transforming the variables and quadratic forms, as described in Appendix C. Appendix C also provides a similar result for multiple ratios of the form $\frac{(\textbf{x}^T \textbf{A} \textbf{x})^l}{(\textbf{x}^T \textbf{B} \textbf{x})^m (\textbf{x}^T \textbf{D} \textbf{x})^n}$.

### Average measures for uniformly distributed selection

With the above theories, average measures can be readily derived as expectations with respect to random $\varvec{\upbeta }$ under the assumption of normality. Previous treatments of evolvability measures (Hansen and Houle 2008; Kirkpatrick 2009) and random skewers analysis (Revell 2007) typically assumed that $\textbf{u}$ is uniformly distributed on the unit hypersphere in the *p*-dimensional space. This condition is entailed in spherical multivariate normal distributions of $\varvec{\upbeta }$: $\varvec{\upbeta } \sim N_p \left( \textbf{0}_p, \sigma ^2 \textbf{I}_p \right)$ for arbitrary positive $\sigma ^2$. This section provides expressions of average measures under this condition. (Note that the normality of $\varvec{\upbeta }$ is not necessary for $\textbf{u}$ to be uniformly distributed; i.e., all results in this section hold as long as the distribution of $\varvec{\upbeta }$ is spherically symmetric.) Expressions for a general normal case $\varvec{\upbeta } \sim N_p \left( \varvec{\upeta }, \varvec{\Sigma } \right)$ are given in Appendix C. Most of the results below can be derived either by applying Proposition 1 or Proposition 4 in Appendix B, or directly as done in Appendix A.

The average evolvability ${\bar{e}}$ is straightforward to obtain:

12 $$\begin{aligned} {\bar{e}}&= {{\,\textrm{E}\,}}_{\textbf{u}}\! \left( \textbf{u}^T \textbf{G} \textbf{u} \right) \nonumber \\&= {{\,\textrm{E}\,}}_{\textbf{u}}\! \left( {{\,\textrm{tr}\,}}\left( \textbf{G} \textbf{u} \textbf{u}^T \right) \right) \nonumber \\&= {{\,\textrm{tr}\,}}\left( \textbf{G} \right) / p, \end{aligned}$$

as derived by Hansen and Houle (2008). This is because ${{\,\textrm{E}\,}}_{\textbf{u}}\! \left( \textbf{u} \textbf{u}^T \right) = \textbf{I}_p / p$ under this condition.

Conditional evolvability $c(\varvec{\upbeta })$ and autonomy $a(\varvec{\upbeta })$ can be nonzero only when $\varvec{\upbeta } \in R \left( \textbf{G} \right)$ (see above). When $\textbf{G}$ is singular, this happens with probability 0 when $\varvec{\upbeta }$ is continuously distributed across the entire space. Hence, the average conditional evolvability ${\bar{c}}$ and the average autonomy ${\bar{a}}$ are 0 when $\textbf{G}$ is singular. Although the following expressions can operationally be applied to singular $\textbf{G}$ by using $\textbf{G}^-$ in place of $\textbf{G}^{-1}$, such a result is invalid because the ratio expressions do not hold when $\varvec{\upbeta } \notin R \left( \textbf{G} \right)$.

When $\textbf{G}$ is nonsingular, the average conditional evolvability ${\bar{c}}$ is:

13 $$\begin{aligned} {\bar{c}}&= {{\,\textrm{E}\,}}_{\varvec{\upbeta }}\! \left( \frac{ \varvec{\upbeta }^T \varvec{\upbeta } }{ \varvec{\upbeta }^T \textbf{G}^{-1} \varvec{\upbeta } } \right) \nonumber \\&= \alpha \sum _{k=0}^{\infty } \frac{ \left( \frac{1}{2} \right) _{k} }{ \left( \frac{p}{2} \right) _{k} } C_{\left[ {k} \right] } \! \left( \textbf{I}_p - \alpha \textbf{G}^{-1} \right) \nonumber \\&= \alpha \sum _{k=0}^{\infty } \frac{ \left( 1 \right) _{k} }{ \left( \frac{p}{2} \right) _{k} } d_{k} \! \left( \textbf{I}_p - \alpha \textbf{G}^{-1} \right) \nonumber \\&= \alpha \, {}_2F_1 \left( \frac{1}{2}, 1; \frac{p}{2} ; \textbf{I}_p - \alpha \textbf{G}^{-1} \right) , \end{aligned}$$

where $\alpha$ is any positive constant that satisfies $0< \alpha < 2 / \lambda _{\max } \left( \textbf{G}^{-1}\right)$ (see Proposition 1), $C_{\left[ {k} \right] } \! \left( \cdot \right)$ are the *k*th top-order zonal polynomials, and ${}_2F_1 \left( a, b; c; \cdot \right)$ is an alternative expression using the hypergeometric function of matrix argument with the three parameters *a*, *b*, and *c* (see Appendix A). When $p = 2$, this expression simplifies to the geometric mean of the two eigenvalues of $\textbf{G}$ as mentioned by Hansen and Houle (2008) (Appendix D).

The average autonomy ${\bar{a}}$ is, when $\textbf{G}$ is nonsingular,

14 $$\begin{aligned} {\bar{a}}&= {{\,\textrm{E}\,}}_{\varvec{\upbeta }}\! \left( \frac{(\varvec{\upbeta }^T \varvec{\upbeta })^2}{\varvec{\upbeta }^T \textbf{G} \varvec{\upbeta } \varvec{\upbeta }^T \textbf{G}^{-1} \varvec{\upbeta }} \right) \nonumber \\&= \alpha _1 \alpha _2 \sum _{i=0}^{\infty } \sum _{j=0}^{\infty } \frac{ \left( \frac{1}{2} \right) _{i+j} }{ \left( \frac{p}{2} \right) _{i+j} } C^{\left[ {i} \right] , \left[ {j} \right] }_{\left[ {i+j} \right] } \! \left( \textbf{I}_p - \alpha _1 \textbf{G} , \textbf{I}_p - \alpha _2 \textbf{G}^{-1} \right) \nonumber \\&= \alpha _1 \alpha _2 \sum _{i=0}^{\infty } \sum _{j=0}^{\infty } \frac{ \left( 1 \right) _{i} \left( 1 \right) _{j} }{ \left( \frac{p}{2} \right) _{i+j} } d_{i,j} \! \left( \textbf{I}_p - \alpha _1 \textbf{G} , \textbf{I}_p - \alpha _2 \textbf{G}^{-1} \right) , \end{aligned}$$

where $\alpha _1$ and $\alpha _2$ are to satisfy $0< \alpha _1 < 2 / \lambda _{\max } \! \left( \textbf{G} \right)$ and $0< \alpha _2 < 2 / \lambda _{\max } \! \left( \textbf{G}^{-1} \right)$. The average integration ${\bar{i}}$ is simply $1 - {\bar{a}}$ (and equals 1 when $\textbf{G}$ is singular).

The average respondability ${\bar{r}}$ is

15 $$\begin{aligned} {\bar{r}}&= {{\,\textrm{E}\,}}_{\varvec{\upbeta }}\! \left( \sqrt{ \frac{\varvec{\upbeta }^T \textbf{G}^2 \varvec{\upbeta }}{\varvec{\upbeta }^T \varvec{\upbeta }} } \right) \nonumber \\&= \alpha ^{-\frac{1}{2}} \sum _{k=0}^{\infty } \frac{ \left( -\frac{1}{2} \right) _{k} \left( \frac{1}{2} \right) _{k} }{ \left( \frac{p}{2} \right) _{k} k! } C_{\left[ {k} \right] } \! \left( \textbf{I}_p - \alpha \textbf{G}^2 \right) \nonumber \\&= \alpha ^{-\frac{1}{2}} \sum _{k=0}^{\infty } \frac{ \left( -\frac{1}{2} \right) _{k} }{ \left( \frac{p}{2} \right) _{k} } d_{k} \! \left( \textbf{I}_p - \alpha \textbf{G}^2 \right) \nonumber \\&= \alpha ^{-\frac{1}{2}} \, {}_2F_1 \left( -\frac{1}{2}, \frac{1}{2}; \frac{p}{2} ; \textbf{I}_p - \alpha \textbf{G}^2 \right) , \end{aligned}$$

where $\alpha$ is to satisfy $0< \alpha < 2 / \lambda _{\max } \! \left( \textbf{G}^2 \right)$ (note that $\lambda _{\max } \! \left( \textbf{G}^2 \right) = \lambda _{\max } \! \left( \textbf{G} \right) ^2$ since $\textbf{G}$ is nonnegative definite). Kirkpatrick (2009) discussed on an equivalent quantity, the average (relative) selection response (${\bar{R}}$ there), but did not provide a closed-form expression for arbitrary $\textbf{G}$. It is possible to show his expression for the special case with complete integration is entailed in the present result (Appendix D).

The average flexibility ${\bar{f}}$ is

16 $$\begin{aligned} {\bar{f}}&= {{\,\textrm{E}\,}}_{\varvec{\upbeta }}\! \left( \frac{\varvec{\upbeta }^T \textbf{G} \varvec{\upbeta }}{\sqrt{ \varvec{\upbeta }^T \varvec{\upbeta } \varvec{\upbeta }^T \textbf{G}^2 \varvec{\upbeta }}} \right) \nonumber \\&= \alpha ^{\frac{1}{2}} \sum _{k=0}^{\infty } \frac{ \left( \frac{1}{2} \right) _{k} \left( \frac{1}{2} \right) _{k+1} }{ \left( \frac{p}{2} \right) _{k+1} k! } C^{\left[ {k} \right] , \left[ {1} \right] }_{\left[ {k+1} \right] } \! \left( \textbf{I}_p - \alpha \textbf{G}^2, \textbf{G} \right) \nonumber \\&= \alpha ^{\frac{1}{2}} \sum _{k=0}^{\infty } \frac{\left( \frac{1}{2} \right) _{k}}{\left( \frac{p}{2} \right) _{k+1}}d_{k,1} \! \left( \textbf{I}_p - \alpha \textbf{G}^2, \textbf{G} \right) , \end{aligned}$$

where $\alpha$ is to satisfy $0< \alpha < 2 / \lambda _{\max } \! \left( \textbf{G}^2 \right)$. In general, ${\bar{f}}$ is well defined even if $\textbf{G}$ is singular, unless $\varvec{\upbeta } \in N \left( \textbf{G} \right)$ with nonzero probability (which of course does not happen here).

The average response difference ${\bar{d}}$ has a form essentially identical to ${\bar{r}}$ except for the argument matrix

17 $$\begin{aligned} {\bar{d}}&= {{\,\textrm{E}\,}}_{\varvec{\upbeta }}\! \left( \sqrt{ \frac{\varvec{\upbeta }^T \left( \textbf{G}_1 - \textbf{G}_2 \right) ^2 \varvec{\upbeta }}{\varvec{\upbeta }^T \varvec{\upbeta }} } \right) \nonumber \\&= \alpha ^{-\frac{1}{2}} \sum _{k=0}^{\infty } \frac{ \left( -\frac{1}{2} \right) _{k} \left( \frac{1}{2} \right) _{k} }{ \left( \frac{p}{2} \right) _{k} k! } C_{\left[ {k} \right] } \! \left( \textbf{I}_p - \alpha \left( \textbf{G}_1 - \textbf{G}_2 \right) ^2 \right) \nonumber \\&= \alpha ^{-\frac{1}{2}} \sum _{k=0}^{\infty } \frac{ \left( -\frac{1}{2} \right) _{k} }{ \left( \frac{p}{2} \right) _{k} } d_{k} \! \left( \textbf{I}_p - \alpha \left( \textbf{G}_1 - \textbf{G}_2 \right) ^2 \right) \nonumber \\&= \alpha ^{-\frac{1}{2}} \, {}_2F_1 \left( -\frac{1}{2}, \frac{1}{2}; \frac{p}{2} ; \textbf{I}_p - \alpha \left( \textbf{G}_1 - \textbf{G}_2 \right) ^2 \right) , \end{aligned}$$

where $\alpha$ is to satisfy $0< \alpha < 2 / \lambda _{\max } \! \left( \left( \textbf{G}_1 - \textbf{G}_2 \right) ^2 \right)$.

The average response correlation ${\bar{\rho }}$ is

18 $$\begin{aligned} {\bar{\rho }}&= {{\,\textrm{E}\,}}_{\varvec{\upbeta }}\! \left( \frac{ \varvec{\upbeta }^T \textbf{G}_1 \textbf{G}_2 \varvec{\upbeta } }{ \sqrt{ \varvec{\upbeta }^T \textbf{G}_1^2 \varvec{\upbeta } \varvec{\upbeta }^T \textbf{G}_2^2 \varvec{\upbeta } } } \right) \nonumber \\&= (\alpha _1 \alpha _2)^{\frac{1}{2}} \sum _{i=0}^{\infty } \sum _{j=0}^{\infty } \frac{ \left( \frac{1}{2} \right) _{i} \left( \frac{1}{2} \right) _{j} \left( \frac{1}{2} \right) _{i+j+1} }{ \left( \frac{p}{2} \right) _{i+j+1} i! j! } \nonumber \\&\quad \cdot C^{\left[ {i} \right] , \left[ {j} \right] , \left[ {1} \right] }_{\left[ {i+j+1} \right] } \! \left( \textbf{I}_p - \alpha _1 \textbf{G}_1^2 , \textbf{I}_p - \alpha _2 \textbf{G}_2^2 , \frac{\textbf{G}_1 \textbf{G}_2 + \textbf{G}_2 \textbf{G}_1}{2} \right) \nonumber \\&= (\alpha _1 \alpha _2)^{\frac{1}{2}} \sum _{i=0}^{\infty } \sum _{j=0}^{\infty } \frac{ \left( \frac{1}{2} \right) _{i} \left( \frac{1}{2} \right) _{j} }{ \left( \frac{p}{2} \right) _{i+j+1} } d_{i,j,1} \! \left( \textbf{I}_p - \alpha _1 \textbf{G}_1^2 , \textbf{I}_p - \alpha _2 \textbf{G}_2^2 , \frac{\textbf{G}_1 \textbf{G}_2 + \textbf{G}_2 \textbf{G}_1}{2} \right) , \end{aligned}$$

where $\alpha _1$ and $\alpha _2$ are to satisfy $0< \alpha _1 < 2 / \lambda _{\max } \! \left( \textbf{G}_1^2 \right)$ and $0< \alpha _2 < 2 / \lambda _{\max } \! \left( \textbf{G}_2^2 \right)$.

### Truncation error

It is seen that the successive terms in the above series decrease in magnitude, thus a partial sum up to certain higher-order terms can in principle be used as an approximation of the exact value of the infinite series. Although each term can be easily evaluated with a recursive algorithm (Bao and Kan 2013; Hillier et al. 2014), accurate numerical evaluation involving higher-order terms is practically a computer-intensive problem. The speed of numerical convergence and computational time will be examined in the next section.

The choice of $\alpha$ is arbitrary within the constraint $0< \alpha < 2 / \lambda _{\max }$ with respect to the relevant matrix (see above), but influences the signs of the terms as well as the speed of convergence. When $0 < \alpha \le 1 / \lambda _{\max }$, all the zonal and invariant polynomials in the above expressions are positive because the argument matrices have only nonnegative eigenvalues. Therefore, all terms in the summations in ${\bar{c}}$ (13), ${\bar{a}}$ (14), ${\bar{f}}$ (16), and ${\bar{\rho }}$ (18) are positive, so truncation results in (slight) underestimation. On the other hand, all terms in ${\bar{r}}$ (15) and ${\bar{d}}$ (17), except those for $k = 0$, are negative in the same condition, so truncation results in overestimation. If $1 / \lambda _{\max }< \alpha < 2 / \lambda _{\max }$, the signs of the polynomials possibly, though not always, fluctuate, rendering the behavior of the series less predictable.

It is possible to derive bounds for the truncation errors in ${\bar{c}}$, ${\bar{r}}$, ${\bar{f}}$, and ${\bar{d}}$ by applying the method of Hillier et al. (2009, theorem 6) with slight modifications, if $\textbf{G}$ is nonsingular and the constants $\alpha$ are taken within $0 < \alpha \le 1 / \lambda _{\max }$. Let ${\bar{c}}_M$, ${\bar{r}}_M$, ${\bar{f}}_M$, and ${\bar{d}}_M$ be the relevant partial sums up to $k = M$. The truncation error bounds are:

19 $$\begin{aligned} 0&\le {\bar{c}} - {\bar{c}}_M \le \frac{\left( 1 \right) _{M+1}}{\left( \frac{p}{2} \right) _{M+1}} \left( \alpha ^{-\frac{p-2}{2}} \left|\textbf{G} \right|^{\frac{1}{2}} - \alpha \sum _{k=0}^{M} d_{k} \! \left( \textbf{I}_p - \alpha \textbf{G}^{-1} \right) \right) , \end{aligned}$$

20 $$\begin{aligned} 0&\ge {\bar{r}} - {\bar{r}}_M \ge \frac{\left( -\frac{1}{2} \right) _{M+1}}{\left( \frac{p}{2} \right) _{M+1}} \left( \alpha ^{-\frac{p+1}{2}} \left|\textbf{G} \right|^{-1} - \alpha ^{-\frac{1}{2}} \sum _{k=0}^{M} d_{k} \! \left( \textbf{I}_p - \alpha \textbf{G}^{2} \right) \right) , \end{aligned}$$

21 $$\begin{aligned} 0&\le {\bar{f}} - {\bar{f}}_M \nonumber \\&\le \frac{\left( \frac{1}{2} \right) _{M+1}}{\left( \frac{p}{2} \right) _{M+2}} \left( \alpha ^{-\frac{p+1}{2}} \left|\textbf{G} \right|^{-1} \frac{ {{\,\textrm{tr}\,}}{ \left( \textbf{G}^{-1} \right) } }{2} - \alpha ^{\frac{1}{2}} \sum _{k=0}^{M} d_{k,1} \! \left( \textbf{I}_p - \alpha \textbf{G}^2 , \textbf{G} \right) \right) , \end{aligned}$$

22 $$\begin{aligned} 0&\ge {\bar{d}} - {\bar{d}}_M \nonumber \\&\ge \frac{\left( -\frac{1}{2} \right) _{M+1}}{\left( \frac{p}{2} \right) _{M+1}} \left( \alpha ^{-\frac{p+1}{2}} \left|\textbf{G}_1 - \textbf{G}_2 \right|^{-1} - \alpha ^{-\frac{1}{2}} \sum _{k=0}^{M} d_{k} \! \left( \textbf{I}_p - \alpha \left( \textbf{G}_1 - \textbf{G}_2 \right) ^{2} \right) \right) , \end{aligned}$$

where the constants $\alpha$ are as in the corresponding definitions (but to satisfy $0 < \alpha \le 1 / \lambda _{\max }$). Unfortunately, the same method are not applicable to ${\bar{a}}$ or ${\bar{\rho }}$. Also, if $\textbf{G}$ (or $\textbf{G}_1 - \textbf{G}_2$ in case of ${\bar{d}}$) is singular, these error bounds do not hold.

Empirically, a larger value of $\alpha$ often improves the speed of convergence, as more eigenvalues of the argument matrix approach 0, but this should be used with caution since the above error bounds are not strictly applicable if $\alpha > 1 / \lambda _{\max }$. To be precise, the error bounds should hold even under this condition provided that all the zonal/invariant polynomials above $k = M$ are nonnegative, but it is not obvious in which case that condition can be shown to be true.

## Numerical evaluation

### Software implementation

There exist two Matlab programs distributed by Raymond M. Kan (https://www-2.rotman.utoronto.ca/~kan/) for evaluating the moments of simple ratios of quadratic forms in normal variables (Hillier et al. 2009, 2014; Bao and Kan 2013), which can be used to evaluate ${\bar{c}}$, ${\bar{r}}$, and ${\bar{d}}$ if a suitable environment is available. Alternatively, these average measures could be evaluated as hypergeometric functions of matrix argument, for which an efficient, general algorithm has been developed by Koev and Edelman (2006) and implemented in the R package HypergeoMat (Laurent 2022). However, that seems relatively inefficient in the present applications where the terms corresponding to all non-top-order partitions can be omitted. None of these can be used to evaluate moments of multiple ratios like ${\bar{a}}$, ${\bar{f}}$, and ${\bar{\rho }}$.

In order to make all above expressions available in the popular statistical software environment R, the present author has developed an independent implementation of the algorithms of Hillier et al. (2014) and Bao and Kan (2013) and published it as the package qfratio on the CRAN repository (Watanabe 2023). This package can be used to evaluate general moments of ratios of quadratic forms in normal variables with arbitrary mean and covariance (under certain constraints for singular cases as discussed in Appendix C.1). It uses RcppEigen (Bates and Eddelbuettel 2013) to effectively execute the computationally intensive recursive algorithms.

### Numerical experiment: methods

Using the new R package, numerical experiments were conducted in a small set of artificial covariance matrices to evaluate behaviors and efficiency of the present series expressions. For each of $p = 5, 20, 50$, and 200, three different eigenvalue conformations were used; two were set to have single large eigenvalues with different magnitudes of integration, $V_\textrm{rel} = 0.1, 0.5$—corresponding to relatively weak and rather strong integration, respectively—and one was set to have a quadratically decreasing series of eigenvalues (for the algorithms see Watanabe (2022) and the R package eigvaldisp (https://github.com/watanabe-j/eigvaldisp)). The matrices with these eigenvalue conformations are referred to as $\textbf{G}_{0.1}$, $\textbf{G}_{0.5}$, and $\textbf{G}_\textrm{Q}$. For each of these, two eigenvector conformations were used so that the resultant covariance matrices were either diagonal matrices of the eigenvalues or correlation matrices with the same eigenvalues constructed with Givens rotations (Davies and Higham 2000). For the one-matrix measures (${\bar{c}}$, ${\bar{r}}$, ${\bar{a}}$, and ${\bar{f}}$), the choice of eigenvectors is inconsequential. For the two-matrix measures (${\bar{d}}$ and ${\bar{\rho }}$), comparisons were made between pairs of matrices with identical and different eigenvectors. The results were also compared to those from the traditional Monte Carlo evaluation using the qfratio package and the delta method approximation of Hansen and Houle (2008, 2009) using new codes.

When possible, an average computational time from 10 runs is recorded for the present series evaluation and Monte Carlo evaluation with 10,000 iterations in each condition. This is except for ${\bar{\rho }}$ in certain conditions with $p \ge 50$ where only a single run was timed for the series evaluation as it did not reach numerical convergence (below). Where a truncation error bound is available, an order of series evaluation *M* was chosen so that the error is below $1.0 \times 10^{-8}$. Where an error bound is unavailable, it was aimed to evaluate the series until the result rounded to the digit of $10^{-9}$ appeared stable. (These values were arbitrarily chosen for benchmarking purposes, and not to be used as a guide for applications.) No formal timing was done for the delta method approximation as it is not computer-intensive. All computational times reported are in elapsed time rather than CPU time. The calculations were executed on a regular desktop PC with Intel^®^ Core i7-8700 CPU and 16 GB RAM, running R version 4.2.3 (R Core Team 2023) with its built-in BLAS. For those problems that required larger computational memory for convergence (${\bar{\rho }}$ with $\textbf{G}_{0.5}$ and $p \ge 20$; see below), another machine with 256 GB RAM was used to calculate accurate values. The codes used in the numerical experiments are provided as Online Resource 1, as well as maintained on a GitHub repository (https://github.com/watanabe-j/avrevol).

### Numerical experiment: results

**Fig. 2:**
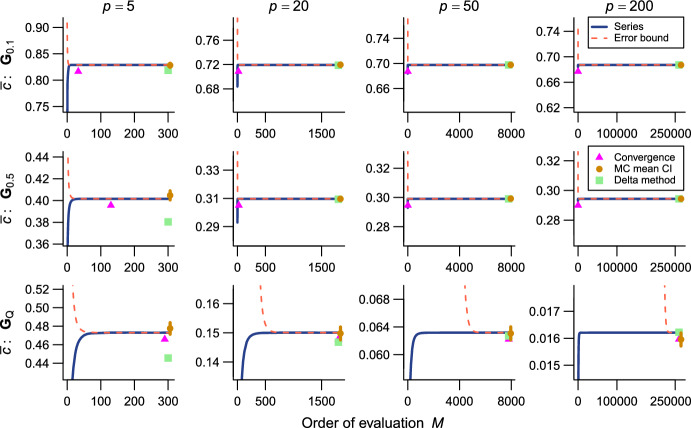
Results of numerical experiments for ${\bar{c}}$. Partial sums from series expressions are shown in blue solid lines, and associated truncation error bounds as deviations from the partial sum are in red broken lines, across varying orders of evaluation *M*. Mean and its 95% confidence interval from a Monte Carlo run with 10,000 iterations are shown in brown circles and vertical bars. Delta method approximations by Hansen and Houle (2008, 2009) are shown in green squares. Rows correspond to eigenvalue conformations (see text) and columns to number of variables *p*. Each panel represents $\pm 10\%$ region of the terminal value of series evaluation

**Fig. 3:**
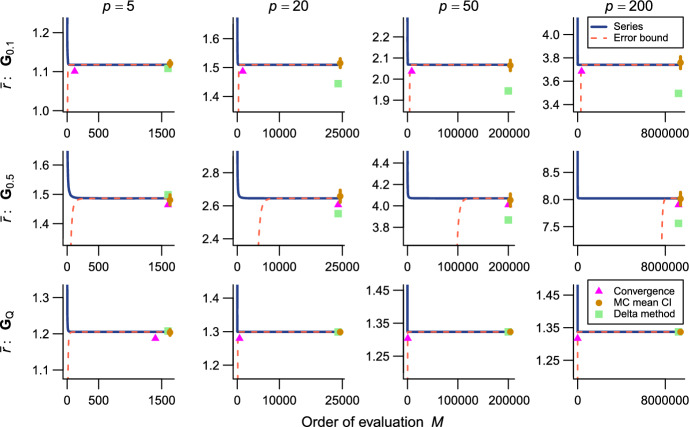
Results of numerical experiments for ${\bar{r}}$. See Fig. 2 for legend

**Fig. 4:**
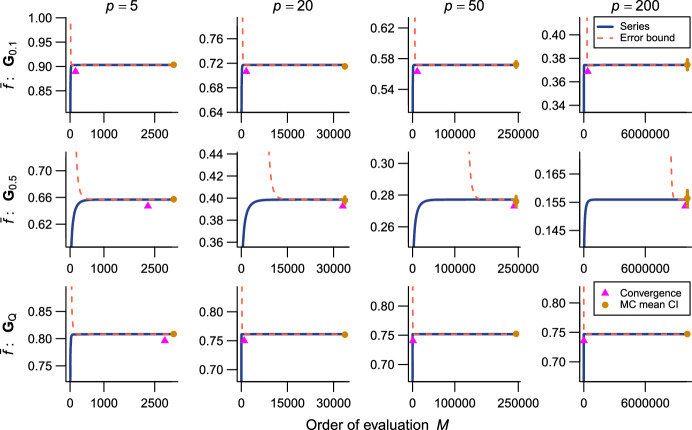
Results of numerical experiments for ${\bar{f}}$. See Fig. 2 for legend. Delta method approximation is not available

**Fig. 5:**
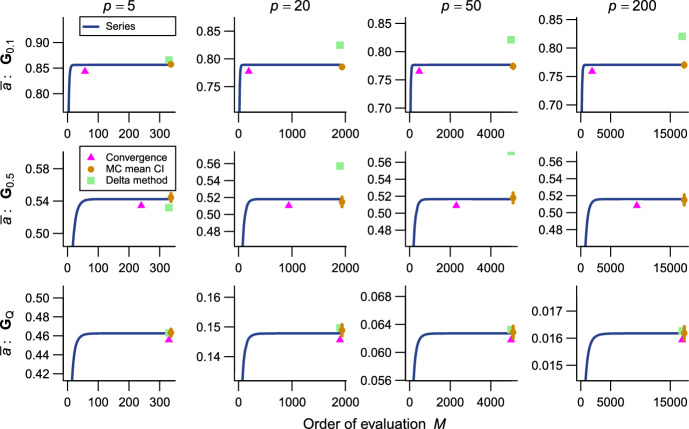
Results of numerical experiments for ${\bar{a}}$. See Fig. 2 for legend. Truncation error bounds are not available. In some results, delta method approximation is too inaccurate to be plotted in the panels

**Fig. 6:**
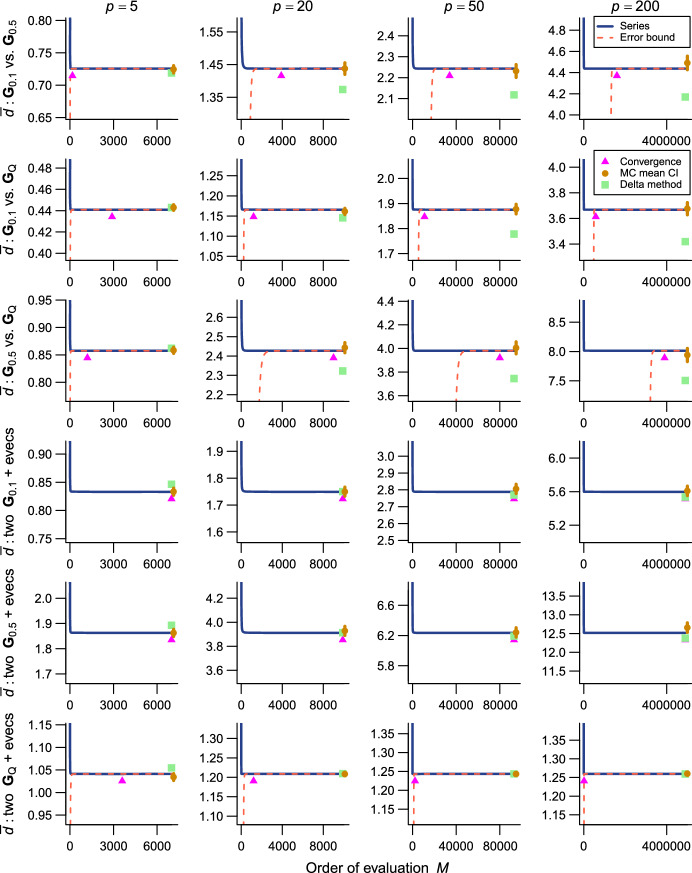
Results of numerical experiments for ${\bar{d}}$. Top three rows are comparisons between covariance matrices with different eigenvalues and same eigenvectors, whereas the bottom three rows are between matrices with same eigenvalues and different eigenvectors (indicated by “+ evecs”). Comparisons between different eigenvalues and different eigenvectors are not shown for space limitations. See Fig. 2 for legend. Error bounds are not available in some same-eigenvalue comparisons because of the singularity of argument matrices (see text)

**Fig. 7:**
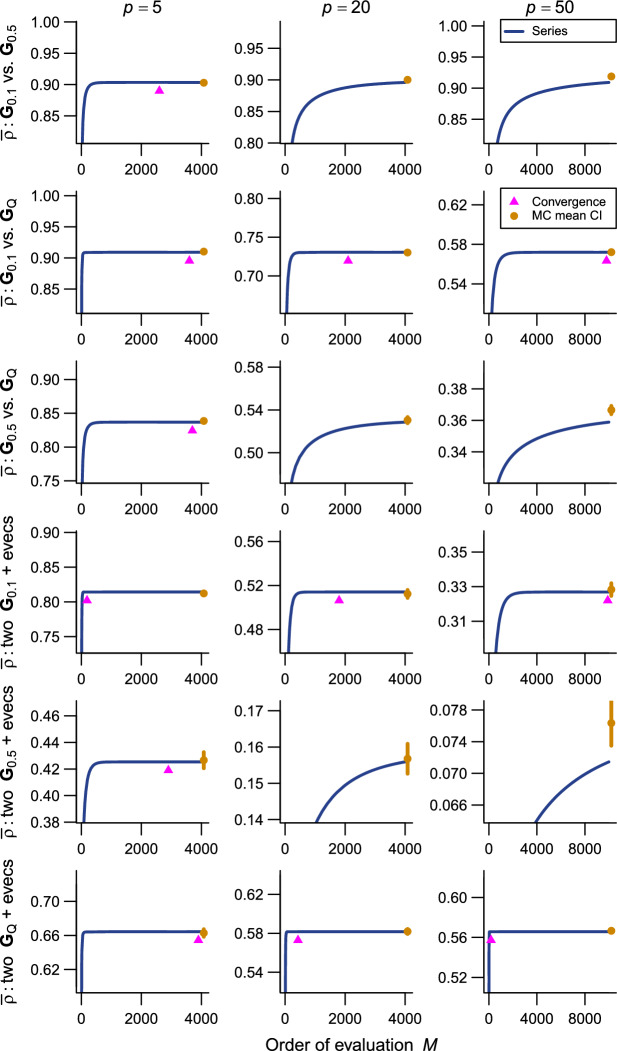
Results of numerical experiments for ${\bar{\rho }}$. Top three rows are comparisons between covariance matrices with different eigenvalues and same eigenvectors, whereas the bottom three rows are between matrices with same eigenvalues and different eigenvectors. Comparisons between different eigenvalues and different eigenvectors are not shown for space limitations. See Fig. 2 for legend. Delta method approximation or error bound is not available

**Fig. 8:**
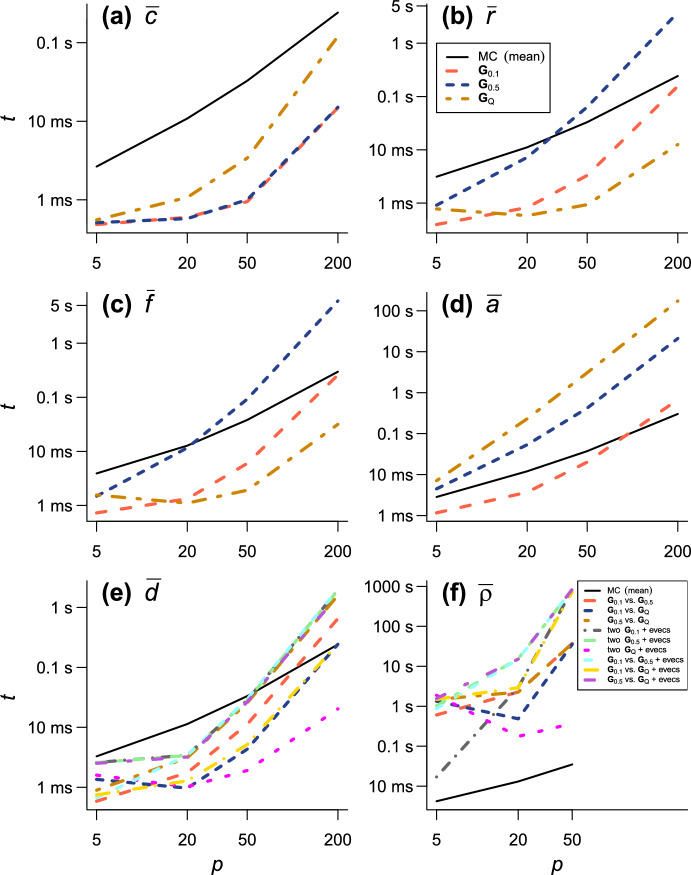
Summary of computational time. **a** conditional evolvability ${\bar{c}}$; **b** respondability ${\bar{r}}$; **c** flexibility ${\bar{f}}$; **d** autonomy ${\bar{a}}$; **e** response difference ${\bar{d}}$; **f** response correlation ${\bar{\rho }}$. Average computational time *t* for each eigenvalue conformation (**a**–**d**; legend in **b**) and combination of matrices (**e**, **f**; legend in **f**) is shown against varying *p*, along with average computational time for Monte Carlo evaluation (MC). “+ evecs” indicates combinations with different eigenvectors. Both axes in log scale

The present series evaluations as implemented in the R package qfratio reached numerical convergence within reasonable computational times in all cases except ${\bar{\rho }}$ with large ($p = 200$) or highly integrated ($\textbf{G}_{0.5}$) matrices (detailed below). The order of evaluation *M* required to accomplish a given level of accuracy varied across evolvability measures and eigenvalue conformations, but in most cases increased with *p* (Figs. 2, 3, 4, 5, 6, 7; Tables 1, 2, 3, 4, 5, 6). The rate of scaling with respect to *p* tended to be more rapid in the series evaluation than in Monte Carlo evaluation, when the required accuracy for the former and the number of iteration for the latter are pre-determined as described above (Fig. 8).

For ${\bar{c}}$, the computational time required for the error bound to numerically converge below $1.0 \times 10^{-8}$ was at most in the order of $\sim$0.1 s and compared favorably with the Monte Carlo evaluation with 10,000 iterations across the entire range of *p* examined (Fig. 8; Table 1). For ${\bar{r}}$ and ${\bar{f}}$, the computational time varied from $\sim$1.5 ms in $p = 5$ to several seconds in $p = 200$, and in some eigenvalue conformations the series evaluation was outcompeted by the Monte Carlo evaluation with a fixed number of iterations in terms of plain computational time (Fig. 8; Tables 2, 3). However, it is to be remembered that results from Monte Carlo evaluations always involve stochastic error, whose standard error (SE) scales only with the inverse square root of the number of iteration (i.e., 100 times as many calculations are required to reduce the SE by the factor of 1/10). It is also notable that the partial sums themselves typically appeared to reach asymptotes much earlier than the error bounds did in these measures (Figs. 2, 3, 4). It took substantially longer time to accurately evaluate ${\bar{a}}$ than the other one-matrix measures, since it involves a double series so that the computational burden scales roughly with $M^2$. Consequently, the computational time required for ${\bar{a}}$ to numerically converge at the order of $10^{-9}$ varied from $\sim$7 ms to $\sim$170 s (Fig. 8; Table 4). Technically, this large computational time was partly because of the necessity to scale individual terms in the evaluation of ${\bar{a}}$ to avoid numerical overflow and underflow.

**Table 1:** Results of numerical experiments for ${\bar{c}}$

Matrix	*p*	*M*	${\bar{c}}_M$	Error bound	$t_\textrm{S}$ (ms)	$t_\textrm{MC}$ (ms)	$\mathrm {SE_{MC}}$	${\hat{c}}_\textrm{HH}$	$\Delta _\textrm{HH}$ (%)
$\textbf{G}_{0.1}$	5	33	0.8289	6.637 $\times$ 10$^{-9}$	0.5	2.8	2.029 $\times$ 10$^{-3}$	0.8187	$-$1.2
	20	19	0.7194	9.622 $\times$ 10$^{-9}$	0.6	10.9	5.355 $\times$ 10$^{-4}$	0.7188	$-$0.1
	50	9	0.6977	9.309 $\times$ 10$^{-9}$	0.9	32.8	2.019 $\times$ 10$^{-4}$	0.6976	0.0
	200	5	0.6872	2.978 $\times$ 10$^{-9}$	14.8	239.2	4.753 $\times$ 10$^{-5}$	0.6872	0.0
$\textbf{G}_{0.5}$	5	130	0.4016	4.687 $\times$ 10$^{-9}$	0.5	2.6	2.138 $\times$ 10$^{-3}$	0.3803	$-$5.3
	20	25	0.3097	8.858 $\times$ 10$^{-9}$	0.6	10.7	2.580 $\times$ 10$^{-4}$	0.3094	$-$0.1
	50	10	0.2991	5.217 $\times$ 10$^{-9}$	1.0	32.8	9.143 $\times$ 10$^{-5}$	0.2991	0.0
	200	5	0.2944	3.512 $\times$ 10$^{-9}$	15.1	242.3	2.077 $\times$ 10$^{-5}$	0.2944	0.0
$\textbf{G}_\textrm{Q}$	5	290	0.4730	7.239 $\times$ 10$^{-9}$	0.6	2.6	3.212 $\times$ 10$^{-3}$	0.4456	$-$5.8
	20	1800	0.1501	6.932 $\times$ 10$^{-9}$	1.1	10.9	1.132 $\times$ 10$^{-3}$	0.1468	$-$2.2
	50	7800	0.0632	8.195 $\times$ 10$^{-9}$	3.4	32.6	4.837 $\times$ 10$^{-4}$	0.0627	$-$0.7
	200	260,000	0.0162	4.445 $\times$ 10$^{-10}$	120.4	243.4	1.233 $\times$ 10$^{-4}$	0.0162	0.1

Tabulated are combinations of eigenvalue conformation and number of variables *p*, order of evaluation *M* required to attain the accuracy specified in text, partial sum from series evaluation ${\bar{c}}_M$ and its error bound, average computational time of series and Monte Carlo evaluations, $t_\textrm{S}$ and $t_\textrm{MC}$, empirical standard error from one Monte Carlo run with 10,000 iterations $\mathrm {SE_{MC}}$, Hansen and Houle’s delta method approximation ${\hat{c}}_\textrm{HH}$, and its deviation from ${\bar{c}}_M$ in percent $\Delta _\textrm{HH}$

**Table 2:** Results of numerical experiments for ${\bar{r}}$

Matrix	*p*	*M*	${\bar{r}}_M$	Error bound	$t_\textrm{S}$ (ms)	$t_\textrm{MC}$ (ms)	$\mathrm {SE_{MC}}$	${\hat{r}}_\textrm{HH}$	$\Delta _\textrm{HH}$ (%)
$\textbf{G}_{0.1}$	5	120	1.1176	–6.518 $\times$ 10$^{-9}$	0.4	3.1	3.897 $\times$ 10$^{-3}$	1.1082	$-$0.8
	20	1300	1.5095	–3.571 $\times$ 10$^{-9}$	0.8	11.0	7.891 $\times$ 10$^{-3}$	1.4443	$-$4.3
	50	8500	2.0677	–9.517 $\times$ 10$^{-9}$	3.3	32.9	1.279 $\times$ 10$^{-2}$	1.9439	$-$6.0
	200	360,000	3.7407	–9.967 $\times$ 10$^{-10}$	156.4	241.1	2.630 $\times$ 10$^{-2}$	3.4955	$-$6.6
$\textbf{G}_{0.5}$	5	1600	1.4865	–8.769 $\times$ 10$^{-9}$	0.9	3.3	8.931 $\times$ 10$^{-3}$	1.4986	0.8
	20	24,000	2.6450	–8.893 $\times$ 10$^{-9}$	7.2	11.1	1.872 $\times$ 10$^{-2}$	2.5521	$-$3.5
	50	200,000	4.0700	–6.271 $\times$ 10$^{-9}$	63.4	33.3	2.966 $\times$ 10$^{-2}$	3.8681	$-$5.0
	200	9,200,000	8.0216	–9.269 $\times$ 10$^{-9}$	3759.2	245.1	6.091 $\times$ 10$^{-2}$	7.5602	$-$5.8
$\textbf{G}_\textrm{Q}$	5	1400	1.2052	–7.504 $\times$ 10$^{-9}$	0.8	3.1	4.079 $\times$ 10$^{-3}$	1.2078	0.2
	20	540	1.2992	–9.765 $\times$ 10$^{-9}$	0.6	11.3	2.585 $\times$ 10$^{-3}$	1.2990	0.0
	50	700	1.3237	–7.742 $\times$ 10$^{-9}$	0.9	32.8	1.737 $\times$ 10$^{-3}$	1.3237	0.0
	200	2100	1.3370	–3.993 $\times$ 10$^{-9}$	12.6	241.3	8.872 $\times$ 10$^{-4}$	1.3370	0.0

See Table 1 for legend

**Table 3:** Results of numerical experiments for ${\bar{f}}$

Matrix	*p*	*M*	${\bar{f}}_M$	Error bound	$t_\textrm{S}$ (ms)	$t_\textrm{MC}$ (ms)	$\mathrm {SE_{MC}}$
$\textbf{G}_{0.1}$	5	160	0.9031	3.628 $\times$ 10$^{-9}$	0.7	3.9	5.258 $\times$ 10$^{-4}$
	20	1600	0.7172	5.471 $\times$ 10$^{-9}$	1.3	12.6	1.459 $\times$ 10$^{-3}$
	50	11,000	0.5718	8.857 $\times$ 10$^{-10}$	6.0	38.4	1.986 $\times$ 10$^{-3}$
	200	380,000	0.3743	1.267 $\times$ 10$^{-9}$	258.9	298.6	2.214 $\times$ 10$^{-3}$
$\textbf{G}_{0.5}$	5	2300	0.6569	6.730 $\times$ 10$^{-9}$	1.5	4.0	1.382 $\times$ 10$^{-3}$
	20	33,000	0.3987	8.228 $\times$ 10$^{-9}$	11.6	12.8	1.626 $\times$ 10$^{-3}$
	50	240,000	0.2772	9.889 $\times$ 10$^{-9}$	92.6	37.9	1.627 $\times$ 10$^{-3}$
	200	9,900,000	0.1559	3.968 $\times$ 10$^{-9}$	6052.1	295.2	1.434 $\times$ 10$^{-3}$
$\textbf{G}_\textrm{Q}$	5	2800	0.8082	9.271 $\times$ 10$^{-9}$	1.6	3.9	1.119 $\times$ 10$^{-3}$
	20	910	0.7614	9.615 $\times$ 10$^{-9}$	1.1	12.9	7.039 $\times$ 10$^{-4}$
	50	930	0.7518	8.400 $\times$ 10$^{-9}$	1.9	38.2	4.640 $\times$ 10$^{-4}$
	200	2400	0.7470	1.679 $\times$ 10$^{-10}$	31.7	298.6	2.364 $\times$ 10$^{-4}$

See Table 1 for legend

**Table 4:** Results of numerical experiments for ${\bar{a}}$

Matrix	*p*	*M*	${\bar{a}}_M$	$t_\textrm{S}$ (ms)	$t_\textrm{MC}$ (ms)	$\mathrm {SE_{MC}}$	${\hat{a}}_\textrm{HH}$	$\Delta _\textrm{HH}$ (%)
$\textbf{G}_{0.1}$	5	57	0.8563	1.2	2.8	1.010 $\times$ 10$^{-3}$	0.8656	1.1
	20	190	0.7893	3.7	11.9	1.826 $\times$ 10$^{-3}$	0.8245	4.5
	50	470	0.7766	20.1	37.4	1.989 $\times$ 10$^{-3}$	0.8210	5.7
	200	1900	0.7705	675.1	295.3	2.069 $\times$ 10$^{-3}$	0.8205	6.5
$\textbf{G}_{0.5}$	5	240	0.5425	4.5	2.8	2.508 $\times$ 10$^{-3}$	0.5319	− 2.0
	20	940	0.5181	53.6	11.9	2.948 $\times$ 10$^{-3}$	0.5572	7.5
	50	2300	0.5165	419.9	37.3	3.003 $\times$ 10$^{-3}$	0.5728	10.9
	200	9400	0.5160	21055.3	305.2	3.028 $\times$ 10$^{-3}$	0.5823	12.9
$\textbf{G}_\textrm{Q}$	5	330	0.4626	7.1	2.8	1.900 $\times$ 10$^{-3}$	0.4631	0.1
	20	1900	0.1478	231.0	12.3	9.823 $\times$ 10$^{-4}$	0.1496	1.2
	50	5000	0.0627	3062.5	37.3	4.552 $\times$ 10$^{-4}$	0.0633	0.9
	200	17,000	0.0162	173985.7	306.8	1.234 $\times$ 10$^{-4}$	0.0163	0.5

See Table 1 for legend

**Table 5:** Results of numerical experiments for ${\bar{d}}$

Matrices	*p*	*M*	${\bar{d}}_M$	Error bound	$t_\textrm{S}$ (ms)	$t_\textrm{MC}$ (ms)	$\mathrm {SE_{MC}}$	${\hat{d}}_\textrm{HH}$	$\Delta _\textrm{HH}$ (%)
$\textbf{G}_{0.1}$ vs. $\textbf{G}_{0.5}$, same evecs	5	170	0.7256	–5.603 $\times$ 10$^{-9}$	0.6	3.2	2.914 $\times$ 10$^{-3}$	0.7189	$-$0.9
	20	3900	1.4375	–8.787 $\times$ 10$^{-9}$	1.8	11.7	9.087 $\times$ 10$^{-3}$	1.3736	$-$4.4
	50	34,000	2.2428	–6.090 $\times$ 10$^{-9}$	11.2	33.0	1.572 $\times$ 10$^{-2}$	2.1176	$-$5.6
	200	1,600,000	4.4371	–1.852 $\times$ 10$^{-9}$	651.5	241.0	3.287 $\times$ 10$^{-2}$	4.1696	$-$6.0
$\textbf{G}_{0.1}$ vs. $\textbf{G}_\textrm{Q}$, same evecs	5	2900	0.4409	–9.244 $\times$ 10$^{-9}$	1.4	3.2	1.372 $\times$ 10$^{-3}$	0.4429	0.5
	20	1200	1.1657	–5.016 $\times$ 10$^{-9}$	1.0	11.4	4.027 $\times$ 10$^{-3}$	1.1456	$-$1.7
	50	11,000	1.8752	–4.953 $\times$ 10$^{-9}$	4.3	33.0	9.865 $\times$ 10$^{-3}$	1.7781	$-$5.2
	200	580,000	3.6679	–2.875 $\times$ 10$^{-9}$	243.1	240.9	2.446 $\times$ 10$^{-2}$	3.4190	$-$6.8
$\textbf{G}_{0.5}$ vs. $\textbf{G}_\textrm{Q}$, same evecs	5	1200	0.8575	–6.347 $\times$ 10$^{-9}$	0.9	3.2	2.907 $\times$ 10$^{-3}$	0.8620	0.5
	20	9000	2.4270	–9.410 $\times$ 10$^{-9}$	3.1	11.2	1.346 $\times$ 10$^{-2}$	2.3229	$-$4.3
	50	80,000	3.9804	–9.196 $\times$ 10$^{-9}$	25.7	33.5	2.607 $\times$ 10$^{-2}$	3.7449	$-$5.9
	200	3,900,000	8.0124	–2.997 $\times$ 10$^{-9}$	1589.6	240.2	5.761 $\times$ 10$^{-2}$	7.5050	$-$6.3
Two $\textbf{G}_{0.1}$, different evecs	5	7000	0.8330	–	2.6	3.4	3.243 $\times$ 10$^{-3}$	0.8465	1.6
	20	9900	1.7493	–	3.4	11.2	8.644 $\times$ 10$^{-3}$	1.7500	0.0
	50	93,000	2.7882	–	28.5	33.2	1.413 $\times$ 10$^{-2}$	2.7693	$-$0.7
	200	4,900,000	5.5980	–	1943.8	242.0	2.892 $\times$ 10$^{-2}$	5.5357	$-$1.1
Two $\textbf{G}_{0.5}$, different evecs	5	7000	1.8627	–	2.6	3.4	7.318 $\times$ 10$^{-3}$	1.8929	1.6
	20	9900	3.9116	–	3.4	11.4	1.937 $\times$ 10$^{-2}$	3.9131	0.0
	50	93,000	6.2347	–	28.4	33.5	3.188 $\times$ 10$^{-2}$	6.1923	$-$0.7
	200	4,900,000	12.5174	–	1938.2	240.5	6.569 $\times$ 10$^{-2}$	12.3783	$-$1.1
Two $\textbf{G}_\textrm{Q}$, different evecs	5	3600	1.0412	–9.597 $\times$ 10$^{-9}$	1.6	3.4	3.909 $\times$ 10$^{-3}$	1.0546	1.3
	20	1200	1.2087	–4.817 $\times$ 10$^{-9}$	1.0	11.5	2.174 $\times$ 10$^{-3}$	1.2095	0.1
	50	2300	1.2433	–3.719 $\times$ 10$^{-9}$	1.9	33.1	1.344 $\times$ 10$^{-3}$	1.2434	0.0
	200	14,000	1.2595	–8.004 $\times$ 10$^{-11}$	20.4	243.9	6.708 $\times$ 10$^{-4}$	1.2595	0.0
$\textbf{G}_{0.1}$ vs. $\textbf{G}_{0.5}$, different evecs	5	450	1.4432	-8.000 $\times$ 10$^{-9}$	0.7	3.2	5.645 $\times$ 10$^{-3}$	1.4478	0.3
	20	9900	2.9662	–9.012 $\times$ 10$^{-9}$	3.4	11.2	1.587 $\times$ 10$^{-2}$	2.8938	$-$2.4
	50	93,000	4.6991	–9.283 $\times$ 10$^{-9}$	29.8	33.2	2.698 $\times$ 10$^{-2}$	4.5298	$-$3.6
	200	4,900,000	9.4026	–5.707 $\times$ 10$^{-9}$	2004.6	242.8	5.638 $\times$ 10$^{-2}$	8.9991	$-$4.3
$\textbf{G}_{0.1}$ vs. $\textbf{G}_\textrm{Q}$, different evecs	5	690	0.9545	–9.978 $\times$ 10$^{-9}$	0.7	3.2	3.271 $\times$ 10$^{-3}$	0.9630	0.9
	20	2200	1.4997	–9.404 $\times$ 10$^{-9}$	1.3	11.3	6.282 $\times$ 10$^{-3}$	1.4564	$-$2.9
	50	13,000	2.0938	–5.509 $\times$ 10$^{-9}$	5.2	33.0	1.160 $\times$ 10$^{-2}$	1.9798	$-$5.4
	200	590,000	3.7798	–9.522 $\times$ 10$^{-9}$	248.9	241.3	2.529 $\times$ 10$^{-2}$	3.5236	$-$6.8
$\textbf{G}_{0.5}$ vs. $\textbf{G}_\textrm{Q}$, different evecs	5	6800	1.5135	–9.579 $\times$ 10$^{-9}$	2.5	3.2	5.735 $\times$ 10$^{-3}$	1.5199	0.4
	20	8600	2.7842	–9.060 $\times$ 10$^{-9}$	3.2	11.6	1.592 $\times$ 10$^{-2}$	2.6625	$-$4.4
	50	82,000	4.2077	–9.025 $\times$ 10$^{-9}$	26.4	33.4	2.843 $\times$ 10$^{-2}$	3.9590	$-$5.9
	200	3,900,000	8.1259	–9.530 $\times$ 10$^{-9}$	1577.7	237.9	5.910 $\times$ 10$^{-2}$	7.6115	$-$6.3

Comparisons were made in pairs of covariance matrices with same and different eigenvectors (evecs). Error bound was unavailable for comparisons between two $\textbf{G}_{0.1}$’s and two $\textbf{G}_{0.5}$’s with different eigenvectors (see text). See Table 1 for further explanations

**Table 6:** Results of numerical experiments for ${\bar{\rho }}$

Matrices	*p*	*M*	${\bar{\rho }}_M$	${\bar{\rho }}_{M'}$	$t_\textrm{S}$ (s)	$t_\textrm{MC}$ (s)	$\mathrm {SE_{MC}}$
$\textbf{G}_{0.1}$ vs. $\textbf{G}_{0.5}$, same evecs	5	2600	0.9034	–	0.60	0.00	6.306 $\times$ 10$^{-4}$
	20	4000	(0.8960)	0.8990	2.27	0.01	7.670 $\times$ 10$^{-4}$
	50	10,000	(0.9092)	0.9192	37.37*	0.03	7.896 $\times$ 10$^{-4}$
$\textbf{G}_{0.1}$ vs. $\textbf{G}_\textrm{Q}$, same evecs	5	3600	0.9090	–	1.34	0.00	7.897 $\times$ 10$^{-4}$
	20	2100	0.7306	–	0.48	0.01	6.966 $\times$ 10$^{-4}$
	50	9800	0.5720	–	35.68	0.04	8.863 $\times$ 10$^{-4}$
$\textbf{G}_{0.5}$ vs. $\textbf{G}_\textrm{Q}$, same evecs	5	3700	0.8369	–	1.47	0.00	1.138 $\times$ 10$^{-3}$
	20	4000	(0.5286)	0.5308	2.26	0.01	1.466 $\times$ 10$^{-3}$
	50	10,000	(0.3589)	0.3657	37.41*	0.04	1.337 $\times$ 10$^{-3}$
Two $\textbf{G}_{0.1}$, different evecs	5	190	0.8143	–	0.02	0.00	1.091 $\times$ 10$^{-3}$
	20	1800	0.5141	–	2.96	0.01	1.831 $\times$ 10$^{-3}$
	50	9900	0.3269	–	830.93	0.03	1.856 $\times$ 10$^{-3}$
Two $\textbf{G}_{0.5}$, different evecs	5	2900	0.4254	–	1.02	0.00	3.076 $\times$ 10$^{-3}$
	20	4000	(0.1559)	0.1585	15.49	0.01	2.098 $\times$ 10$^{-3}$
	50	10,000	(0.0715)	0.0767	845.44*	0.03	1.457 $\times$ 10$^{-3}$
Two $\textbf{G}_\textrm{Q}$, different evecs	5	3900	0.6644	–	1.88	0.00	2.239 $\times$ 10$^{-3}$
	20	430	0.5816	–	0.18	0.01	1.295 $\times$ 10$^{-3}$
	50	180	0.5657	–	0.37	0.03	7.993 $\times$ 10$^{-4}$
	200	110	0.5581	–	4.37	0.18	4.017$\times$ 10$^{-4}$
$\textbf{G}_{0.1}$ vs. $\textbf{G}_{0.5}$, different evecs	5	2700	0.5904	–	0.87	0.00	2.056 $\times$ 10$^{-3}$
	20	4000	(0.2835)	0.2856	15.03	0.01	1.936 $\times$ 10$^{-3}$
	50	10000	(0.1532)	0.1584	845.41*	0.04	1.543 $\times$ 10$^{-3}$
$\textbf{G}_{0.1}$ vs. $\textbf{G}_\textrm{Q}$, different evecs	5	3500	0.7293	–	1.50	0.00	1.729 $\times$ 10$^{-3}$
	20	1800	0.5435	–	2.91	0.01	1.647 $\times$ 10$^{-3}$
	50	9200	0.4293	–	717.31	0.04	1.684 $\times$ 10$^{-3}$
$\textbf{G}_{0.5}$ vs. $\textbf{G}_\textrm{Q}$, different evecs	5	3600	0.5276	–	1.57	0.00	2.636 $\times$ 10$^{-3}$
	20	4000	(0.2979)	0.3001	15.13	0.01	1.939 $\times$ 10$^{-3}$
	50	10000	(0.2009)	0.2077	845.67*	0.03	1.524 $\times$ 10$^{-3}$

Parentheses around ${\bar{\rho }}_M$ denote values that did not reach numerical convergence with specified *M*; for these conditions, more accurate values with larger *M* were calculated on a separate machine and are shown for reference (${\bar{\rho }}_{M'}$)—this calculation was not timed. For $p = 200$, it was infeasible to apply the series evaluation in most combinations for excessive computational memory required. See Table 5 for further explanations

Asterisks after $t_\textrm{S}$ denote times from single runs rather than 10 runs

For two-matrix comparisons, ${\bar{d}}$ behaved similarly to ${\bar{r}}$ with which it shares the same functional form (Fig. 6; Table 5). In some comparisons between matrices with the same eigenvalues and different eigenvectors, the error bound of ${\bar{d}}$ could not be calculated because the argument matrix ($\left( \textbf{G}_1 - \textbf{G}_2 \right) ^2$) was singular due to the artificial conformations of eigenvalues in $\textbf{G}_{0.1}$ and $\textbf{G}_{0.5}$ where the trailing eigenvalues are identical in magnitude. Series evaluation of ${\bar{\rho }}$ took substantially more computational time and memory than the other measures. For $p \ge 20$, the series could not be evaluated until numerical convergence in any comparisons that involve $\textbf{G}_{0.5}$ due to excessively large memory requirement for the 16 GB RAM machine used here for main calculations, at least when the accuracy in the order of $10^{-9}$ is aimed at (Fig. 7; Table 6 involves accurate values from a larger machine). This is apparently because $\textbf{G}_{0.5}^2$ has rather small eigenvalues, which translate into extremely large magnitudes of higher-order terms, $d_{i,j,1}$ in (18). For $p = 200$, accurate series evaluation of ${\bar{\rho }}$ seemed infeasible in most combinations for too much computational memory required. The only exception in the present test cases is the comparison between two $\textbf{G}_\textrm{Q}$’s with different eigenvectors where the series converged fairly rapidly (Table 6).

As expected, the Monte Carlo evaluation always yielded an unbiased estimate with the confidence intervals having the intended nominal coverage of the converged series where available. As implemented in the qfratio package, iterations in the order of $10^4$ can be executed within a second, so might be favored over the new series expression when the computational burden is a constraint and stochastic error is tolerated. Interestingly, relative accuracies of the series and Monte Carlo evaluations showed a qualitatively similar pattern of variation across eigenvalue conformations for a given average measure and *p*; the Monte Carlo evaluation tended to yield a large SE when the series evaluation or its error bound was slow to converge (Figs. 2, 3, 4, 5, 6, 7). In other words, eigenvalue conformations seem to largely determine the accuracy at which average measures can be empirically evaluated.

The delta method approximations by Hansen and Houle (2008, 2009) can be evaluated almost instantaneously (from $\sim$0.1 to $\sim$7 ms in $p = 5$ and 200), as they are straightforward arithmetic operations once eigenvalues of the argument matrix are obtained. For ${\bar{c}}$ in large *p*, they showed notable empirical accuracy (Fig. 2; Table 1) (see also Hansen and Houle 2008). In many other cases, however, they yielded inaccurate values, often deviating from the converged series by >5%, and it is even difficult to discern a consistent trend in the directions of errors (Figs. 3, 5, 6; Tables 2, 4, 5). Except in the former cases, it is rather questionable when those approximations can be reliably used.

## Discussion

The present paper derived new expressions for average evolvability measures using results on the moments of ratios of quadratic forms in normal variables. These series expressions are in theory exact, although for practical evaluation summation must be truncated, yielding truncation error. A great advantage in the present approach is that a hard upper bound for the truncation error is known for some of the measures: namely, average conditional evolvability ${\bar{c}}$, average respondability ${\bar{r}}$, average flexibility ${\bar{f}}$, and average response difference ${\bar{d}}$ (above). This is unlike the confidence intervals from Monte Carlo evaluations, which always involve some uncertainty. In addition, empirically speaking, evaluation of most of the average measures, excepting average autonomy ${\bar{a}}$ and average response correlation ${\bar{\rho }}$, can be faster than Monte Carlo evaluation for modest-sized problems (e.g., $p \le 50$), depending on the accuracy aimed at. The accuracy and speed of the series evaluation will facilitate practical studies that involve quantification and/or comparison of evolvability or integration using average measures, for which the Monte Carlo evaluation or delta method approximation has traditionally been used.

As a practical guide for calculating average measures, the series evaluation is recommended whenever numerical convergence can be reached within reasonable computational time and memory. Its feasibility largely depends on the eigenvalues of $\textbf{G}$, but it is always easy to check whether convergence has been reached by inspecting a profile of the partial sum and by using error bounds when available. When the computational time and/or memory is a constraint (for ${\bar{a}}$ and ${\bar{\rho }}$ in large *p* with many small eigenvalues), then an alternative will be Monte Carlo evaluation, which is adequate because the evolvability measures considered herein have finite second moments (Appendix B). The delta method approximations as proposed by Hansen and Houle (2008, 2009) can yield rather inaccurate values with hardly predictable errors (above), thus will not remain a viable option in estimating average measures. This is perhaps except when even the (typically sub-second) computational burden of the Monte Carlo evaluation is a bottleneck, e.g., when one has large Bayesian samples of $\textbf{G}$. However, a primary problem in the delta method remains that, although the method empirically works well in certain situations (e.g., ${\bar{c}}$ in large *p*), there is no theoretical guarantee for it to work well in general situations; the accuracy always need to be assessed by another method of evaluation.

It should be remembered that, in empirical analyses, $\textbf{G}$ is almost always estimated with uncertainty, so that estimates of (average) evolvability measures involve error and potentially bias (Houle and Meyer 2015; Grabowski and Porto 2017). Although uncertainty may partially be incorporated in a Bayesian sampling of $\textbf{G}$ (Aguirre et al. 2014) or a parametric-bootstrap-like sampling with multivariate normal approximation (Houle and Meyer 2015), these methods will not by themselves deal with potential estimation bias. The present series expressions are not free from this problem, which need to be addressed in future investigations. It might be possible to derive sampling moments of the (average) measures under simple sampling conditions (e.g., Wishartness), as was recently done for eigenvalue dispersion indices of covariance and correlation matrices (Watanabe 2022). A potentially promising fact is that the expectations of zonal and invariant polynomials of Wishart matrices are known (Constantine 1963; Davis 1980). It remains to be done to derive equivalent results to polynomials in more complicated functions of random matrices like the ones appearing in the present expressions. Apart from that, simulation-based approaches would also be useful in obtaining rough estimates of sampling error and bias (Haber 2011; Grabowski and Porto 2017). In any case, the present expressions will greatly facilitate future investigations into sampling properties of average measures as they provide quasi-exact values given (estimates of) $\textbf{G}$, unlike the approximate methods used in previous studies.

Although the present paper dealt with six evolvability measures, this is not to endorse all of them. Some of these measures may be disfavored from a biological or other theoretical ground. For example, Hansen and Houle (2008) criticized the use of (average) response correlation ${\bar{\rho }}$ in the random skewers analysis for its insensitivity to potentially different magnitudes of response vectors and recommended (average) response difference ${\bar{d}}$ as an alternative. (To be fair, Cheverud (1996) argued for the use of ${\bar{\rho }}$ by claiming that there tends to be a large sampling bias in the magnitude of response vectors from empirical covariance matrices, but that remains to be justified.) Rohlf (2017) also pointed out difficulties in interpreting ${\bar{\rho }}$ without knowledge on the eigenvalues and eigenvectors of the matrices compared, and recommended simply using those quantities for comparing matrices. The present author refrains from going into details on biological validity or utility of individual evolvability measures, because they will ultimately depend on the scope and context of individual studies as well as the nature of the data analyzed.

It seems worth repeating one of Rohlf’s (2017) insightful caveats here. Rohlf (2017) considered the angle between response vectors (arc cosine of response correlation $\rho$) from two $\textbf{G}$ matrices in bivariate traits and empirically showed that the angle can have a bimodal distribution when $\varvec{\upbeta }$ is distributed uniformly on the unit circle. The interpretation of the mean of such a multimodal distribution will not be straightforward, although, at least as empirically observed, the multimodality may not be a universal phenomenon (see Machado et al. 2018). At present, the possibility remains that other evolvability measures can have multimodal distributions as well. Generally speaking, it is known that the distribution of the ratio of quadratic forms $\textbf{x}^T \textbf{A} \textbf{x} / \textbf{x}^T \textbf{B} \textbf{x}$, where $\textbf{B}$ is positive definite, has different functional forms across intervals bordered by eigenvalues of $\textbf{B}^{-1} \textbf{A}$ (Hillier 2001; Forchini 2002, 2005), around which distinct density peaks can potentially be observed. Formal evaluation of the conditions for multimodality seems rather tedious (if tractable at all) and is not pursued herein. It should at least be borne in mind that the average measures are insensitive to the potential multimodality and that, generally speaking, a scalar summary index like them can mask those nuanced aspects of the underlying distribution and the multivariate covariance structure.

The present paper suggested slight modifications for some evolvability measures to accommodate potentially singular $\textbf{G}$ matrices. As originally defined by Hansen and Houle (2008), conditional evolvability *c* and autonomy *a* (and integration *i*) could not be evaluated for singular $\textbf{G}$ as they required matrix inversion. This restriction can be released by using the generalized inverse as done above. Conditional evolvability *c* and autonomy *a* are zero unless $\varvec{\upbeta } \in R \left( \textbf{G} \right)$. The corresponding average measures (${\bar{c}}$ and ${\bar{a}}$) are also zero unless the same condition is satisfied with nonzero probability (i.e., when $\varvec{\upbeta }$ is continuously distributed, its distribution is to be restricted to $R \left( \textbf{G} \right)$), as partially surmised by Hansen and Houle (2008). The new expressions enable to calculate *c* (4), *a* (6), *i* and their averages (C30), (C31) even when $\textbf{G}$ is singular, as long as $\varvec{\upbeta } \in R \left( \textbf{G} \right)$. Biologically speaking, the singularity of $\textbf{G}$ represents the presence of absolute genetic constraint, where no genetic variance is available in certain directions. Although detection of absolute constraint in empirical systems requires different frameworks than evolvability measures (Mezey and Houle 2005; Hine and Blows 2006; Meyer and Kirkpatrick 2008; Pavlicev et al. 2009), the present development will facilitate investigations into systems with absolute constraints. These will also involve, e.g., shape variables that lack variation in certain directions in the full (nominal) trait space (see below). However, it is necessary to distinguish the singularity in a true $\textbf{G}$ matrix and that in its empirical estimates ($\hat{\textbf{G}}$, say) due to small sample size. It should be viewed with much caution whether evolvability measures can be meaningfully applied to the latter, because $R \left( \hat{\textbf{G}} \right)$ as a space fluctuates due to random sampling when the sample size is not large enough to span the entire $R \left( \textbf{G} \right)$.

Previous treatments of evolvability measures (Hansen and Houle 2008; Kirkpatrick 2009; Marroig et al. 2009; Bolstad et al. 2014) and random skewers (Revell 2007) almost exclusively considered the simple condition where selection gradients are spherically distributed, $\varvec{\upbeta } \sim N_p (\textbf{0}_p, \sigma ^2 \textbf{I}_p)$, or uniformly distributed on a unit hypersphere. As detailed in Appendix C, the present results can be extended to a more general condition where the selection gradient has an arbitrary normal distribution, $\varvec{\upbeta } \sim N_p (\varvec{\upeta }, \varvec{\Sigma })$. These extended results can potentially be useful when interest is in characterizing and comparing evolvability of populations under a certain directional and/or correlated selection regime. Of course, the original expressions of evolvability measures for a fixed $\varvec{\upbeta }$ can be used when the selection is considered deterministic, so the utility of the new extended expressions will be in when the selection is considered random but potentially directional and/or correlated.

Chevin (2013) theoretically investigated the rate of adaptation (change in log average fitness across generations) in the same condition where $\varvec{\upbeta }$ has a general normal distribution. He notably showed that, when stabilizing selection is assumed to be weak, the rate of adaptation is approximately equal to $\varvec{\upbeta }^T \textbf{G} \varvec{\upbeta }$. He called this quantity “evolvability”, ascribing it to Hansen and Houle (2008), and discussed at length on its interpretation. However, that quantity should be distinguished from the evolvability *e* as defined by Hansen et al. (2003b) and Hansen and Houle (2008), which is standardized by the norm of the selection gradient $\varvec{\upbeta }$.^4^ This standardization can also be seen as projection of the selection gradients onto a unit hypersphere (Mardia and Jupp 1999); directional and correlational selections can be expressed as concentration of density on the hypersphere. Accordingly, Chevin’s quantity and the present expressions for evolvability measures under general normal selection regimes have different interpretations and utility. The former would be more appropriate as a measure of absolute rate of adaptation, whereas the latter seems to be more appropriate for characterizing $\textbf{G}$ matrices independently of the magnitude of selection.

The present expressions for a general normal case would be particularly useful when the selection gradients are assumed to be restricted to a certain subspace rather than distributed across the entire trait space, i.e., when $\varvec{\Sigma }$ is singular. Such restriction can potentially arise from biological reasons, but probably are more often encountered from structural constraints in certain types of traits, e.g., shape variables. When the support of the (nominal) traits is restricted to a certain subspace, it would be sensible to assume that selection is also restricted to the same subspace (e.g., when geometric shapes are concerned, it is senseless to consider the components of selection corresponding to geometric rotation or translocation). Along with the accommodation of potentially singular $\textbf{G}$ matrices, this extension allows for application of average measures to various types of traits encountered in the current evolutionary biology. Overall, the present development will facilitate theoretical and empirical investigations into the evolution of multivariate characters, by providing accurate means to quantify and compare various aspects of evolvability, integration, and/or constraint.

### Supplementary Information

Below is the link to the electronic supplementary material.

Supplementary file 1 (7z 9 KB)

## Data Availability

Not applicable.

## Appendix A Zonal and invariant polynomials

This paper evaluates the expectations of ratios of quadratic forms using the zonal and invariant polynomials, mainly following Smith (1989) and Hillier et al. (2009, 2014). These polynomials are widely used in the literature of multivariate distribution theory, primarily to integrate out components affected by orthogonal rotations from functions of random matrices. To be precise, they are not strictly required to derive results relevant to the present applications (see Bao and Kan 2013), but allow for a conceptually elegant derivation and provide alternative interpretations of some of the results as hypergeometric functions of matrix argument. A brief introduction to these mathematical toolkits is provided in this section, as they appear to have attained little use in the biological literature. For more details, readers are directed to, e.g., James (1964), Muirhead (1982, 2006), Takemura (1984), Gross and Richards (1987), Mathai et al. (1995), and Chikuse (2003, appendix A).

### A.1 Notations

In this section, $\textbf{Y}$ denotes a $p \times p$ symmetric matrix with eigenvalues $y_1, y_2, \dots , y_p$. $\textbf{H}$ denotes a $p \times p$ orthogonal matrix, and the space of $p \times p$ orthogonal matrices is denoted by *O*(*p*).

For indexing polynomials, (ordered) partitions $\kappa$ of positive integers *k* are used; $\kappa {=} (k_1, k_2, \dots )$, where $k_i$ are nonnegative integers that satisfy $k_1 {\ge } k_2 \ge \dots$ and $\sum _i k_i = k$. For instance, the possible partitions of $k = 4$ are $(4), (3, 1), (2, 2), (2, 1, 1), \text {and } (1, 1, 1, 1)$. The partition of *k* with a single nonzero integer part is called the top-order partition of *k*, and is denoted by $\left[ {k} \right]$.

The Pochhammer’s symbol is

A1 $$\begin{aligned} \left( a \right) _{k}&= a (a + 1) \dots (a + k - 1) \nonumber \\&= \frac{\Gamma (a + k)}{\Gamma (a)}, \quad a \ne 0, -1, -2, \dots , \end{aligned}$$

where ${\Gamma (\cdot )}$ is the gamma function, with the understanding $\left( a \right) _{0} = 1$. A multivariate extension $\left( a \right) _\kappa$ (indexed by a partition) is defined as

A2 $$\begin{aligned} \left( a \right) _\kappa&= \prod _{i=1}^m \left( a - \frac{1}{2}(i - 1) \right) _{k_i}. \end{aligned}$$

### A.2 Definitions of zonal polynomials

The zonal polynomials are a certain class of polynomials in matrix entries which has several useful properties. The primary definition of the zonal polynomials comes from the group representation theory (James 1960, 1961, 1964), which tends to be too abstract to comprehend for anyone new to the concept. There are alternative, more operational definitions of the zonal polynomials (Muirhead 1982; Takemura 1984), which would be more approachable to most biologists. However, those alternative definitions do not generalize into the invariant polynomials of multiple matrix arguments, which are also relevant to the present application. For this reason, the representation-theoretic definition is briefly outlined herein, omitting technical details. See Gross and Richards (1987) for a readable and sufficiently comprehensive introduction to the topic.

Consider the vector space $V_k$ of homogeneous polynomials $\varphi (\textbf{Y})$ of degree *k* in the elements of the symmetric matrix $\textbf{Y}$. The transformation with a $p \times p$ invertible matrix $\textbf{L}$,

$$\begin{aligned} \textbf{Y} \rightarrow \textbf{L} \textbf{Y} \textbf{L}^T, \end{aligned}$$

transforms the vector space by

$$\begin{aligned} \varphi \rightarrow T(\textbf{L})(\varphi ) : (T(\textbf{L})(\varphi ))(\textbf{Y}) = \varphi \left( \textbf{L}^{-1} \textbf{Y} {\textbf{L}^{-1}}^T \right) . \end{aligned}$$

(Technically, $T(\textbf{L})$ here is a representation, which abstracts the transformation of the right-hand side by a linear transformation.) It is known that the vector space $V_k$ decomposes into a direct sum of irreducible invariant subspaces $V_\kappa$ as

$$\begin{aligned} V_k = \bigoplus _\kappa V_\kappa , \end{aligned}$$

where the direct summation runs over all partitions $\kappa$ of *k* into not more than *p* parts. By $V_\kappa$ being invariant, it is meant that $T(\textbf{L}) V_\kappa \subset V_\kappa$ for any invertible $\textbf{L}$, and by them being irreducible invariant, it is meant that they do not contain any proper invariant subspace by themselves. It is further known that each $V_\kappa$ contains a unique one-dimensional subspace that is invariant under $\textbf{Y} \rightarrow \textbf{H} \textbf{Y} \textbf{H}^T$ (restricting the above transformation to $\textbf{L} \in O(p)$). Then, $\left[ {{\,\textrm{tr}\,}}\left( \textbf{Y} \right) \right] ^k \in V_k$ has a unique decomposition

A3 $$\begin{aligned} \left[ {{\,\textrm{tr}\,}}\left( \textbf{Y} \right) \right] ^k = \sum _\kappa C_{\kappa } \! \left( \textbf{Y} \right) \end{aligned}$$

into some polynomials $C_{\kappa } \! \left( \textbf{Y} \right) \in V_\kappa$ which satisfy $C_{\kappa } \! \left( \textbf{Y} \right) = C_{\kappa } \! \left( \textbf{H} \textbf{Y} \textbf{H}^T \right)$. These polynomials $C_{\kappa } \! \left( \textbf{Y} \right)$ are called the zonal polynomials corresponding to the partitions $\kappa$. In short, the zonal polynomials of degree *k* constitute a basis of the subspace of $V_k$ that is invariant under the transformation $\textbf{Y} \rightarrow \textbf{H} \textbf{Y} \textbf{H}^T$, each corresponding to the irreducible subspace represented by a partition $\kappa$ of *k*. In terms of (A3), they can be regarded as a generalization of powers of scalars into matrices.

These polynomials can be scaled by arbitrary constants, and there exist several different normalizations in the literature. The normalization used in (A3), with all coefficients for $C_{\kappa } \! \left( \textbf{Y} \right)$ being unity, is primarily for convenience of notation.

### A.3 Basic properties of zonal polynomials

A consequence of the above definition with (A3) is that $C_{\kappa } \! \left( \textbf{Y} \right)$ are polynomials in the eigenvalues $y_1, \dots , y_p$ of $\textbf{Y}$ (note that ${{\,\textrm{tr}\,}}\left( \textbf{Y} \right) = {{\,\textrm{tr}\,}}\left( \textbf{H} \textbf{Y} \textbf{H}^T \right) = \sum _i y_i$). It also entails that they are symmetric against permutation of the eigenvalues. Alternatively, or equivalently, they can be expressed as polynomials in the traces of matrix powers ${{\,\textrm{tr}\,}}\left( \textbf{Y}^j \right)$ ($= \sum _i y_i^j$). Unfortunately, no closed-form expressions are known for the zonal polynomials, except for a few special cases, so the coefficients in these polynomials in general need to be determined recursively (see Muirhead 1982, 2006).

The zonal polynomials are originally defined for symmetric matrices, but the definition can be extended. If $\textbf{X}$ is positive definite and $\textbf{Y}$ is symmetric, the eigenvalues of $\textbf{X} \textbf{Y}$ and $\textbf{X}^{1/2} \textbf{Y} \textbf{X}^{1/2}$ are the same. Therefore, a zonal polynomial of $\textbf{X} \textbf{Y}$ is defined as $C_{\kappa } \! \left( \textbf{X} \textbf{Y} \right) = C_{\kappa } \! \left( \textbf{X}^{1/2} \textbf{Y} \textbf{X}^{1/2} \right)$.

Properties of the zonal polynomials have been extensively investigated (Constantine 1963; James 1964; Muirhead 1982). Elementary properties involve the following:

- $C_{\kappa } \! \left( a \textbf{Y} \right) = a^k C_{\kappa } \! \left( \textbf{Y} \right)$ holds for any constant *a*.
- If the number of parts in $\kappa$ exceeds the rank of $\textbf{Y}$, $C_{\kappa } \! \left( \textbf{Y} \right) = 0$ (Muirhead 1982, corollary 7.2.4(i)).
- If $\textbf{Y}$ is positive definite, $C_{\kappa } \! \left( \textbf{Y} \right) > 0$ (Muirhead 1982, corollary 7.2.4(ii)).
- $C_{\kappa } \! \left( \textbf{Y} \right) = C_{\kappa } \! \left( {{\,\textrm{diag}\,}}\left( \textbf{Y}, \textbf{0} \right) \right)$, where the right-hand side is with respect to a block diagonal matrix with a square matrix of 0’s of arbitrary dimension.

One of the most important properties of the zonal polynomials is the following integral identity.

#### Proposition 2

(James (1960)) Let $\textbf{A}$ and $\textbf{Y}$ be $p \times p$ symmetric matrices. Then,

A4 $$\begin{aligned} \int _{O(p)} C_{\kappa } \! \left( \textbf{A} \textbf{H} \textbf{Y} \textbf{H}^T \right) (\textrm{d}\textbf{H}) = \frac{ C_{\kappa } \! \left( \textbf{A} \right) C_{\kappa } \! \left( \textbf{Y} \right) }{ C_{\kappa } \! \left( \textbf{I}_m \right) }, \end{aligned}$$

where $(\textrm{d}\textbf{H})$ is the normalized invariant measure on *O*(*p*) that satisfies $\int _{O(p)} (\textrm{d}\textbf{H}) = 1$.

Accessible proofs are given in Muirhead (1982, theorem 7.2.5) and Gross and Richards (1987, proposition 5.5).

### A.4 Top-order zonal polynomials

The zonal polynomials corresponding to the top-order partition, $[k] = (k)$, are called the top-order zonal polynomials and appear frequently in the multivariate distribution theory. An explicit expression of the top-order zonal polynomials is (James 1964, (123)):

A5 $$\begin{aligned} C_{\left[ {k} \right] } \! \left( \textbf{Y} \right) = \frac{k!}{\left( \frac{1}{2} \right) _{k}} \sum _{\sum _j i_j j = k} \left[ \prod _{j=1}^{p} \frac{ 1 }{i_j !} \left( \frac{ s_j }{2 j} \right) ^{i_j} \right] , \end{aligned}$$

where $s_j = \sum _{i=1}^p y_i^j = {{\,\textrm{tr}\,}}\left( \textbf{Y}^j \right)$ and the summation is over all such combinations of the nonnegative integers $i_j$ that satisfy $\sum _{j = 0}^k i_j j = i_1 + 2 i_2 + 3 i_3 + \dots = k$. An alternative expression is given in Gross and Richards (1987, (6.8.1)).

For the identity matrix $\textbf{I}_p$, the expression simplifies as:

A6 $$\begin{aligned} C_{\left[ {k} \right] } \! \left( \textbf{I}_p \right)&= \frac{\left( \frac{p}{2} \right) _k}{\left( \frac{1}{2} \right) _k} . \end{aligned}$$

### A.5 Hypergeometric functions of matrix argument

With the zonal polynomials as a generalization of powers into matrices, the hypergeometric functions of matrix argument are defined as

A7 $$\begin{aligned} {}_mF_n \left( a_1, \dots , a_m ; b_1, \dots , b_n ; \textbf{Y} \right) = \sum _{k=0}^{\infty } \sum _{\kappa } \frac{ \left( a_1 \right) _{\kappa } \dots \left( a_m \right) _{\kappa } }{ \left( b_1 \right) _{\kappa } \dots \left( b_n \right) _{\kappa } } \frac{ C_{\kappa } \! \left( \textbf{Y} \right) }{ k! }, \end{aligned}$$

where the summation $\sum _{\kappa }$ is across all ordered partitions $\kappa$ of *k*, and $\left( x \right) _{\kappa }$ is defined as in (A2). Conditions for convergence are as follows: (i) when $m \le n$, the series converges for all $\textbf{Y}$; (ii) when $m = n + 1$, the series converges for all $\Vert \textbf{Y} \Vert < 1$, where $\Vert \textbf{Y} \Vert$ is the maximum of the absolute values of the eigenvalues; (iii) when $m > n + 1$, the series diverges for all $\textbf{Y} \ne 0$, unless the series terminates (when any $a_i$ is a negative integer). The following paragraphs list a few results relevant to the present applications. More extensive treatments on this topic can be found in, e.g., Muirhead (1982), Gross and Richards (1987), and Chikuse (2003).

If any of $a_i$ is 1/2, the series can be expressed as a weighted sum of top-order zonal polynomials, as $\left( 1/2 \right) _{\kappa } = 0$ unless $\kappa = [k]$ (see (A2)):

A8 $$\begin{aligned} {}_{m+1}F_n \left( a_1, \dots , a_m, \frac{1}{2} ; b_1, \dots , b_n ; \textbf{Y} \right) = \sum _{k=0}^{\infty } \frac{ \left( a_1 \right) _{k} \dots \left( a_m \right) _{k} \left( \frac{1}{2} \right) _{k} }{ \left( b_1 \right) _{k} \dots \left( b_n \right) _{k} } \frac{ C_{\left[ {k} \right] } \! \left( \textbf{Y} \right) }{ k! }. \end{aligned}$$

Analogous to the scalar equivalent, ${}_0F_0$ is an exponential series but about the trace:

A9 $$\begin{aligned} {}_0F_0 \left( \textbf{X} \right)&= \sum _{k=0}^{\infty } \sum _{\kappa } \frac{ C_{\kappa } \! \left( \textbf{Y} \right) }{k!} \nonumber \\&= \sum _{k=0}^{\infty } \frac{ \left( {{\,\textrm{tr}\,}}{\textbf{Y}} \right) ^k }{k!} \nonumber \\&= {{\,\textrm{etr}\,}}\left( {\textbf{Y}} \right) . \end{aligned}$$

Provided that all eigenvalues of $\textbf{Y}$ are less than 1 in absolute values, ${}_1F_0$ is a generalization of the binomial series (Muirhead 1982, corollary 7.3.5):

A10 $$\begin{aligned} {}_1F_0 \left( a ; \textbf{Y} \right)&= \sum _{k=0}^{\infty } \sum _{\kappa } \left( a \right) _{\kappa } \frac{ C_{\kappa } \! \left( \textbf{Y} \right) }{k!} \nonumber \\&= |\textbf{I}_m - \textbf{Y} |^{-a}, \quad \Vert \textbf{Y} \Vert < 1. \end{aligned}$$

### A.6 Invariant polynomials of multiple matrix arguments

The invariant polynomials of multiple matrix arguments provide some extension of the zonal polynomials into multiple matrices (Davis 1979, 1980; Chikuse 1980; Chikuse and Davis 1986a). Accessible introductions to the topic are found in, e.g., Chikuse and Davis (1986b) and Mathai et al. (1995, appendix).

Let $V_{k_1, \dots , k_r} = V_{k_1} \otimes \dots \otimes V_{k_r}$ be the vector space of polynomials of degree $k_1, \dots , k_r$ in the elements of $p \times p$ symmetric matrices $\textbf{Y}_1, \dots , \textbf{Y}_r$, respectively. Consider the following simultaneous transformation with a $p \times p$ invertible matrix $\textbf{L}$:

$$\begin{aligned} \textbf{Y}_1 \rightarrow \textbf{L} \textbf{Y}_1 \textbf{L}^T, \dots , \textbf{Y}_r \rightarrow \textbf{L} \textbf{Y}_r \textbf{L}^T. \end{aligned}$$

First noting the subspaces in $V_{k_1, \dots , k_r}$ that are invariant against this transformation and then by decomposing them into irreducible ones, one has the following decomposition:

$$\begin{aligned} V_{k_1, \dots , k_r}\left( \textbf{Y}_1, \dots , \textbf{Y}_r \right)&= \bigoplus _{\kappa _1, \dots , \kappa _r} \lbrace V_{\kappa _1} \left( \textbf{Y}_1 \right) \otimes \dots \otimes V_{\kappa _r} \left( \textbf{Y}_r \right) \rbrace \\&= \bigoplus _{\kappa _1, \dots , \kappa _r} \bigoplus _{\Phi } V_{\Phi }^{\kappa _1, \dots , \kappa _r} \left( \textbf{Y}_1, \dots , \textbf{Y}_r \right) . \end{aligned}$$

Here, $V_{\kappa _i}$ are analogous to $V_\kappa$ discussed above but for $\textbf{Y}_i$, and $V_{\Phi }^{\kappa _1, \dots , \kappa _r}$ are irreducible invariant subspaces indexed by $\Phi$ and $\kappa _1, \dots , \kappa _r$. $\Phi$ are certain ordered partitions of 2*f*, with $f = k_1 + \dots + k_r$, into not more than *p* parts, whose range is determined by $\kappa _1, \dots , \kappa _r$. $\kappa _i$ run across partitions of $k_i$, $i = 1, \dots , r$, into not more than *p* parts. It is known that only those $V_{\Phi }^{\kappa _1, \dots , \kappa _r}$ with partitions taking the form $\Phi = 2 \phi$, where $\phi$ is a partition of *f*, contain a subspace invariant under the simultaneous transformation $\textbf{Y}_1 \rightarrow \textbf{H} \textbf{Y}_1 \textbf{H}^T, \dots , \textbf{Y}_r \rightarrow \textbf{H} \textbf{Y}_r \textbf{H}^T$, which is one-dimensional. The corresponding polynomials are called the invariant polynomials, $C^{\kappa _1, \dots , \kappa _r}_{\phi } \! \left( \textbf{Y}_1, \dots , \textbf{Y}_r \right)$, indexed by $\kappa _1, \dots , \kappa _r$ and $\phi \in \kappa _1 \cdots \kappa _r$. (There is a potential issue of non-uniqueness, but this is not concerned in most applications).

Explicit forms of the invariant polynomials are even less obvious than the zonal polynomials, but they are polynomials in matrix traces of the form ${{\,\textrm{tr}\,}}\left( \textbf{Y}_1^{a_1} \textbf{Y}_2^{a_2} \dots \textbf{Y}_r^{a_r} \right)$, so that the total degrees in elements of $\textbf{Y}_1, \dots , \textbf{Y}_r$ are $k_1, \dots , k_r$, respectively.

An important generalization of Proposition 2 enabled by the invariant polynomials is the following (see also Mathai et al. 1995, theorem A.3).

#### Proposition 3

(Davis (1980), Chikuse (1980)) Let $\textbf{A}_1, \dots , \textbf{A}_r$ and $\textbf{Y}_1, \dots , \textbf{Y}_r$ be $p \times p$ symmetric matrices, and $\kappa _1, \dots , \kappa _r$ be ordered partitions of $k_1, \dots , k_r$ into not more than *p* parts.

A11 $$\begin{aligned}{} & {} \int _{O(p)} \prod _{i=1}^r C_{\kappa _i} \! \left( \textbf{A}_i \textbf{H} \textbf{Y}_i \textbf{H}^T \right) (\textrm{d}\textbf{H}) \nonumber \\{} & {} = \sum _{\phi \in \kappa _1 \cdots \kappa _r} \frac{ C^{\kappa _1, \dots , \kappa _r}_{\phi } \! \left( \textbf{A}_1, \dots , \textbf{A}_r \right) C^{\kappa _1, \dots , \kappa _r}_{\phi } \! \left( \textbf{Y}_1, \dots , \textbf{Y}_r \right) }{ C_{\phi } \! \left( \textbf{I}_p \right) }, \end{aligned}$$

where the summation runs across all permissible partitions $\phi$ of $f = k_1 + \dots + k_r$ corresponding to $\kappa _1, \dots , \kappa _r$.

One notable corollary from this result is (Davis 1980; Chikuse 1980)

A12 $$\begin{aligned} C^{\kappa _1, \dots , \kappa _r}_{\phi } \! \left( \textbf{A}, \dots , \textbf{A} \right) = \frac{ C^{\kappa _1, \dots , \kappa _r}_{\phi } \! \left( \textbf{I}_p, \dots , \textbf{I}_p \right) }{ C_{\phi } \! \left( \textbf{I}_p \right) } C_{\phi } \! \left( \textbf{A} \right) . \end{aligned}$$

The top-order invariant polynomials, in which $\phi = \left[ {f} \right]$, the top-order partition of *f*, are of particular importance here. This partition occurs only when $\kappa _i = \left[ {k_i} \right]$ for all *i*. An explicit form of the top-order invariant polynomials is (Chikuse 1987; Hillier et al. 2009):

A13 $$\begin{aligned} C^{\left[ {k_1} \right] , \dots , \left[ {k_r} \right] }_{ \left[ {f} \right] } \! \left( \textbf{Y}_1, \dots , \textbf{Y}_r \right)&= \frac{\prod _{i=1}^r k_i !}{ \left( \frac{1}{2} \right) _{f} } d_{k_1, \dots , k_r} . \end{aligned}$$

Here, $d_{k_1, \dots , k_r}$ is defined as:

A14 $$\begin{aligned} d_{k_1, \dots , k_r}&= \sum _{\begin{array}{c} \sum _{i_l} i_l \nu _{i_1, \dots , i_r} = k_l \\ l = 1, \dots , r \end{array}} \left[ \prod _{i_1 = 0}^{k_1} \dots \prod _{i_r = 0}^{k_r} \frac{ 1 }{ \nu _{i_1, \dots , i_r}!} \left( \frac{ p_{i_1, \dots , i_r} }{ 2 \sum _j i_j } \right) ^{\nu _{i_1, \dots , i_r}} \right] , \end{aligned}$$

where $i_j$ run from 0 to $k_j$ (but at least one of $i_j$ is nonzero), and $\nu _{i_1, \dots , i_r}$ are nonnegative integers; these collectively satisfy the *r* linear constraints $\sum _{\nu _1 = 0}^{k_1} \dots \sum _{\nu _r = 0}^{k_r} i_l \nu _{i_1, \dots , i_r} = k_l$ for $l = 1, \dots , r$.^5^ And $p_{i_1, \dots , i_r}$ are the coefficients for $\prod _{j=1}^r t_j^{i_j}$ in the expansion of the following:

A15 $$\begin{aligned} {{\,\textrm{tr}\,}}\left[ \left( t_1 \textbf{Y}_1 + \dots + t_r \textbf{Y}_r \right) ^{\sum _j i_j} \right] . \end{aligned}$$

Note that (A13) simplifies into (A5) when $r = 1$. In addition, it holds that (Chikuse 1987)

A16 $$\begin{aligned} C^{\left[ {k_1} \right] , \dots , \left[ {k_r} \right] }_{\left[ {f} \right] } \! \left( \textbf{I}_m, \dots , \textbf{I}_m \right) = C_{\left[ {f} \right] } \! \left( \textbf{I}_m \right) , \end{aligned}$$

regardless of individual values of $k_i$ (see also (A6)).

### A.7 Example application to evolvability measures

As a demonstration for how above mathematical toolkits act in the present problem of evaluating moments of (multiple) ratios of quadratic forms, an expression of the average autonomy ${\bar{a}}$ is derived here, under the simplest case that $\varvec{\upbeta } \sim N_p(\textbf{0}_p, \sigma ^2 \textbf{I}_p)$, where $\sigma ^2$ is an arbitrary positive constant, and $\textbf{G}$ is nonsingular. The derivation here is a simplified version of that in Smith (1989). When $\textbf{G}$ is singular, the approach of Bao and Kan (2013) can be used instead.

As discussed in the text, $\textbf{u}$ is uniformly distributed on the unit hypersphere $S^{p - 1}$ under this assumption. Thus, the average autonomy ${\bar{a}}$, from the definition in (5), is to be evaluated as the following integral:

A17 $$\begin{aligned} {\bar{a}} = \int _{S^{p - 1}} \left( \textbf{u}^T \textbf{G} \textbf{u} \right) ^{-1} \left( \textbf{u}^T \textbf{G}^{-1} \textbf{u} \right) ^{-1} (\textrm{d} \textbf{u}), \end{aligned}$$

where $(\textrm{d} \textbf{u})$ denotes the normalized uniform measure on $S^{p - 1}$ (see, e.g. Muirhead 1982). To evaluate this, consider the following transformation:

A18 $$\begin{aligned} \left( \textbf{u}^T \textbf{G} \textbf{u} \right) ^{-1}&= \alpha _1 \left( 1 - \textbf{u}^T \left( \textbf{I}_p - \alpha _1 \textbf{G} \right) \textbf{u} \right) ^{-1} \nonumber \\&= \alpha _1 \sum _{k=0}^\infty \left( \textbf{u}^T \left( \textbf{I}_p - \alpha _1 \textbf{G} \right) \textbf{u} \right) ^k \nonumber \\&= \alpha _1 \sum _{k=0}^\infty \sum _{\kappa } C_{\kappa } \! \left( \left( \textbf{I}_p - \alpha _1 \textbf{G} \right) \textbf{u} \textbf{u}^T \right) , \end{aligned}$$

where $\alpha _1$ is a positive constant that satisfy $0< \alpha _1 < 2 / \lambda _{\max } \! \left( \textbf{G} \right)$ (so that $|\textbf{u}^T \left( \textbf{I}_p - \alpha _1 \textbf{G} \right) \textbf{u} |< 1$), the second equation is from the fact $(1 - x)^{-1} = \sum _{k=0}^\infty x^k$ when $|x |< 1$, and the last equation is from (A3) and the fact $\textbf{u}^T \left( \textbf{I}_p - \alpha _1 \textbf{G} \right) \textbf{u} = {{\,\textrm{tr}\,}}\left( \left( \textbf{I}_p - \alpha _1 \textbf{G} \right) \textbf{u} \textbf{u}^T \right)$. Note that (A18) can be written as ${}_1F_0 \left( 1; \left( \textbf{I}_p - \alpha _1 \textbf{G} \right) \textbf{u} \textbf{u}^T \right)$ from (A10) and is uniformly convergent under the assumptions. (This series does not converge when $\textbf{G}$ is singular.) By similar transformations, $\left( \textbf{u}^T \textbf{G}^{-1} \textbf{u} \right) ^{-1} = \alpha _2 \sum _{l=0}^\infty \sum _{\lambda } C_{\lambda } \! \left( \left( \textbf{I}_p - \alpha _2 \textbf{G}^{-1} \right) \textbf{u} \textbf{u}^T \right)$, where $0< \alpha _2 < 2 / \lambda _{\max } \! \left( \textbf{G}^{-1} \right)$. Then, the above integral becomes:

A19 $$\begin{aligned} {\bar{a}}&= \alpha _1 \alpha _2 \int _{S^{p - 1}} \sum _{k=0}^\infty \sum _{l=0}^\infty \sum _{\kappa } \sum _{\lambda } C_{\kappa } \! \left( \left( \textbf{I}_p - \alpha _1 \textbf{G} \right) \textbf{u} \textbf{u}^T \right) \nonumber \\&\quad \cdot C_{\lambda } \! \left( \left( \textbf{I}_p - \alpha _2 \textbf{G}^{-1} \right) \textbf{u} \textbf{u}^T \right) (\textrm{d} \textbf{u}). \end{aligned}$$

Here, term-by-term integration is permissible because the series is uniformly convergent (see also Bao and Kan 2013).

Note that integration of $\textbf{u}$ over $S^{p - 1}$ is equivalent to that of $\textbf{H}$ over *O*(*p*) (e.g., Mardia and Jupp 1999), whence the change of variables $\textbf{u} = \textbf{H} \textbf{e}$ is possible, where $\textbf{e} = (1, 0, \dots 0)^T$ (or $\textbf{e}$ could be any unit vector). By denoting $\textbf{e} \textbf{e}^T = \textbf{E}$, the integral is now

A20 $$\begin{aligned} {\bar{a}}&= \alpha _1 \alpha _2 \sum _{k=0}^\infty \sum _{l=0}^\infty \sum _{\kappa } \sum _{\lambda } \int _{O(p)} C_{\kappa } \! \left( \left( \textbf{I}_p - \alpha _1 \textbf{G} \right) \textbf{H} \textbf{E} \textbf{H}^T \right) \nonumber \\&\quad \cdot C_{\lambda } \! \left( \left( \textbf{I}_p - \alpha _2 \textbf{G}^{-1} \right) \textbf{H} \textbf{E} \textbf{H}^T \right) (\textrm{d} \textbf{H}) \nonumber \\&= \alpha _1 \alpha _2 \sum _{k=0}^\infty \sum _{l=0}^\infty \sum _{\kappa } \sum _{\lambda } \sum _{\phi \in \kappa \cdot \lambda } \frac{ C^{\kappa , \lambda }_{\phi } \! \left( \textbf{I}_p - \alpha _1 \textbf{G}, \textbf{I}_p - \alpha _2 \textbf{G}^{-1} \right) C^{\kappa , \lambda }_{\phi } \! \left( \textbf{E}, \textbf{E} \right) }{ C_{\phi } \! \left( \textbf{I}_p \right) }, \end{aligned}$$

by applying Proposition 3. Furthermore, using (A12), $C^{\kappa , \lambda }_{\phi } \! \left( \textbf{E}, \textbf{E} \right) = C^{\kappa , \lambda }_{\phi } \! \left( \textbf{I}_p, \textbf{I}_p \right) C_{\phi } \! \left( \textbf{E} \right) / C_{\phi } \! \left( \textbf{I}_p \right)$. However, $C_{\phi } \! \left( \textbf{E} \right)$ is nonzero only if $\phi = \left[ {k + l} \right]$, the top-order partition, because $\textbf{E}$ has only one nonzero eigenvalue, which is unity. Hence, the summations for $\kappa , \lambda , \phi$ disappear, leaving only the terms corresponding to the top-order partitions, $\left[ {k} \right] , \left[ {l} \right] , \left[ {k + l} \right]$. Also note that, from (A16), $C^{\left[ {k} \right] , \left[ {l} \right] }_{\left[ {k + l} \right] } \! \left( \textbf{I}_p, \textbf{I}_p \right) / C_{\left[ {k + l} \right] } \! \left( \textbf{I}_p \right) = 1$. The integral finally becomes

A21 $$\begin{aligned} {\bar{a}}&= \alpha _1 \alpha _2 \sum _{k=0}^\infty \sum _{l=0}^\infty \frac{ C^{\left[ {k} \right] , \left[ {l} \right] }_{\left[ {k + l} \right] } \! \left( \textbf{I}_p - \alpha _1 \textbf{G}, \textbf{I}_p - \alpha _2 \textbf{G}^{-1} \right) }{ C_{\left[ {k + l} \right] } \! \left( \textbf{I}_p \right) }. \end{aligned}$$

Inserting (A6) yields (14) in the text. The expressions for all average measures presented in the main text under the same assumption can be derived in a similar manner.

### A.8 Generating functions for top-order zonal and invariant polynomials

For practical evaluation of top-order zonal and invariant polynomials, it is more efficient to handle $d_{k_1, \dots , k_r}$ in (A13) and (A14) than the polynomials themselves. Chikuse (1987) showed that the same $d_{k_1, \dots , k_r} \! \left( \textbf{A}_1 , \dots , \textbf{A}_r \right)$ appear as coefficients in the following, which is the joint moment generating function of $\textbf{x}^T \textbf{A}_1 \textbf{x} / 2, \dots , \textbf{x}^T \textbf{A}_r \textbf{x} / 2$ for $\textbf{x} \sim N_p (\textbf{0}_p, \textbf{I}_p)$:

A22 $$\begin{aligned} \left| \textbf{I}_p - t_1 \textbf{A}_1 - \dots - t_r \textbf{A}_r \right|^{-\frac{1}{2}} = \sum _{k_1 = 0}^{\infty } \dots \sum _{k_r = 0}^{\infty } d_{k_1, \dots , k_r} \! \left( \textbf{A}_1 , \dots , \textbf{A}_r \right) t_1^{k_1} \dots t_r^{k_r}. \end{aligned}$$

Chikuse (1987) and Hillier et al. (2009, 2014) presented a few recursive algorithms to evaluate $d_{k_1, \dots , k_r}$.

Bao and Kan (2013) and Hillier et al. (2014) presented further improvements for evaluating moments of ratios of quadratic forms in potentially noncentral normal variables, $\textbf{x} \sim N_p (\varvec{\upmu }, \textbf{I}_p)$. Following those papers, the coefficients $h_{k_1, k_2}, {\tilde{h}}_{k_1; k_2}, {\tilde{h}}_{k_1; k_2, k_3}$ that appear in the following expansions are used in the present paper:

A23 $$\begin{aligned}&\left| \textbf{I}_p - t_1 \textbf{A}_1 - t_2 \textbf{A}_2 \right|^{-\frac{1}{2}} \exp \left( \frac{ (1 - t_1 - t_2) \varvec{\upmu }^T \left( \textbf{I}_p - t_1 \textbf{A}_1 - t_2 \textbf{A}_2 \right) ^{-1} \varvec{\upmu } - \varvec{\upmu }^T \varvec{\upmu } }{2} \right) \nonumber \\&\quad = \sum _{k_1 = 0}^{\infty } \sum _{k_2 = 0}^{\infty } h_{k_1, k_2} \! \left( \textbf{A}_1 , \textbf{A}_2 \right) t_1^{k_1} t_2^{k_2}, \end{aligned}$$

A24 $$\begin{aligned}&\left| \textbf{I}_p - t_1 \textbf{A}_1 - t_2 \textbf{A}_2 \right|^{-\frac{1}{2}} \exp \left( \frac{ (1 - t_2) \varvec{\upmu }^T \left( \textbf{I}_p - t_1 \textbf{A}_1 - t_2 \textbf{A}_2 \right) ^{-1} \varvec{\upmu } - \varvec{\upmu }^T \varvec{\upmu } }{2} \right) \nonumber \\&\quad = \sum _{k_1 = 0}^{\infty } \sum _{k_2 = 0}^{\infty } {\tilde{h}}_{k_1; k_2} \! \left( \textbf{A}_1 ; \textbf{A}_2 \right) t_1^{k_1} t_2^{k_2}, \end{aligned}$$

A25 $$\begin{aligned}&\left| \textbf{I}_p - t_1 \textbf{A}_1 - t_2 \textbf{A}_2 - t_3 \textbf{A}_3 \right|^{-\frac{1}{2}} \nonumber \\&\quad \cdot \exp \left( \frac{ (1 - t_2 - t_3) \varvec{\upmu }^T \left( \textbf{I}_p - t_1 \textbf{A}_1 - t_2 \textbf{A}_2 - t_3 \textbf{A}_3 \right) ^{-1} \varvec{\upmu } - \varvec{\upmu }^T \varvec{\upmu } }{2} \right) \nonumber \\&\quad = \sum _{k_1 = 0}^{\infty } \sum _{k_2 = 0}^{\infty } \sum _{k_3 = 0}^{\infty } {\tilde{h}}_{k_1; k_2, k_3} \! \left( \textbf{A}_1 ; \textbf{A}_2 , \textbf{A}_3 \right) t_1^{k_1} t_2^{k_2} t_3^{k_3}. \end{aligned}$$

(A23) and (A24) are from Bao and Kan (2013), and (A25) is a slight extension from (A24) used in appendices B and C. All these simplify to (A22) when $\varvec{\upmu } = \textbf{0}_p$. Note that the first arguments in ${\tilde{h}}_{k_1; k_2}$ and ${\tilde{h}}_{k_1; k_2, k_3}$ are not interchangeable with the other arguments, unlike those in $d_{k_1, k_2}$ or $h_{k_1, k_2}$.

## Appendix B Moment of multiple ratio of quadratic forms

Among the average evolvability measures mentioned in the text, the expressions for ${\bar{e}}$, ${\bar{c}}$, ${\bar{r}}$, and ${\bar{d}}$ can be directly derived from Proposition 1. For ${\bar{a}}$, ${\bar{f}}$, and ${\bar{\rho }}$, an expression is required for the expectation of the form $\frac{(\textbf{x}^T \textbf{A} \textbf{x})^l}{(\textbf{x}^T \textbf{B} \textbf{x})^m (\textbf{x}^T \textbf{D} \textbf{x})^n}$; it suffices here to concern integer-valued *l*. Smith (1989) derived such an expression for $\textbf{B}, \textbf{D}$ being positive definite. However, it is possible to derive the following, equivalent expression with a slightly general condition that $\textbf{B}, \textbf{D}$ being nonnegative definite, following a similar line of arguments as in Bao and Kan (2013).

### Proposition 4

Let $\textbf{x} \sim N_p \left( \varvec{\upmu }, \textbf{I}_p \right)$, $\textbf{A}$ be a symmetric $p \times p$ matrix, $\textbf{B}, \textbf{D}$ be symmetric, nonnegative definite $p \times p$ matrices, *l* be a positive integer, and *m*, *n* be positive real numbers. When the expectation of $\frac{(\textbf{x}^T \textbf{A} \textbf{x})^l}{(\textbf{x}^T \textbf{B} \textbf{x})^m (\textbf{x}^T \textbf{D} \textbf{x})^n}$ exists, it is

B26 $$\begin{aligned}&{{\,\textrm{E}\,}}\! \left( \frac{(\textbf{x}^T \textbf{A} \textbf{x})^l}{(\textbf{x}^T \textbf{B} \textbf{x})^m (\textbf{x}^T \textbf{D} \textbf{x})^n} \right) \nonumber \\&\quad = 2^{l - m - n} \alpha _{\textbf{B}}^{m} \alpha _{\textbf{D}}^{n} l! e^{ -\frac{ \varvec{\upmu }^T \varvec{\upmu } }{2} } \sum _{i=0}^{\infty } \sum _{j=0}^{\infty } \sum _{k=0}^{\infty } \frac{ \left( m \right) _{i} \left( n \right) _{j} \Gamma \left( \frac{p}{2} + l - m - n + k \right) }{ 2^k \left( \frac{1}{2} \right) _{k} \Gamma \left( \frac{p}{2} + l + i + j + k \right) } \nonumber \\&\quad \quad \cdot \frac{\left( \frac{1}{2} \right) _{i+j+k+l} }{ i! j! k! } C^{\left[ {i} \right] , \left[ {j} \right] , \left[ {k} \right] , \left[ {l} \right] }_{\left[ {i+j+k+l} \right] } \! \left( \textbf{I}_p - \alpha _{\textbf{B}} \textbf{B} , \textbf{I}_p - \alpha _{\textbf{D}} \textbf{D} , \varvec{\upmu } \,\,\varvec{\upmu }^T , \textbf{A} \right) \end{aligned}$$

B27 $$\begin{aligned}&\quad = 2^{l - m - n} \alpha _{\textbf{B}}^{m} \alpha _{\textbf{D}}^{n} l! e^{ -\frac{ \varvec{\upmu }^T \varvec{\upmu } }{2} } \sum _{i=0}^{\infty } \sum _{j=0}^{\infty } \sum _{k=0}^{\infty } \frac{ \left( m \right) _{i} \left( n \right) _{j} \Gamma \left( \frac{p}{2} + l - m - n + k \right) }{ 2^k \left( \frac{1}{2} \right) _{k} \Gamma \left( \frac{p}{2} + l + i + j + k \right) } \nonumber \\&\quad \quad \cdot d_{i, j, k, l} \! \left( \textbf{I}_p - \alpha _{\textbf{B}} \textbf{B} , \textbf{I}_p - \alpha _{\textbf{D}} \textbf{D} , \varvec{\upmu }\,\, \varvec{\upmu }^T , \textbf{A} \right) \end{aligned}$$

B28 $$\begin{aligned}&\quad = 2^{l - m - n} \alpha _{\textbf{B}}^{m} \alpha _{\textbf{D}}^{n} l! \frac{ \Gamma \left( \frac{p}{2} + l - m - n \right) }{ \Gamma \left( \frac{p}{2} + l \right) } \nonumber \\&\quad \quad \cdot \sum _{i=0}^{\infty } \sum _{j=0}^{\infty } \frac{ \left( m \right) _{i} \left( n \right) _{j} }{ \left( \frac{p}{2} + l \right) _{i + j} } {\tilde{h}}_{l; i, j} \! \left( \textbf{A} ; \textbf{I}_p - \alpha _{\textbf{B}} \textbf{B} , \textbf{I}_p - \alpha _{\textbf{D}} \textbf{D} \right) , \end{aligned}$$

where $\alpha _{\textbf{B}}, \alpha _{\textbf{D}}$ are to satisfy $0< \alpha _{\textbf{B}} < 2 / \lambda _{\max } \! \left( \textbf{B} \right)$ and $0< \alpha _{\textbf{D}} < 2 / \lambda _{\max } \! \left( \textbf{D} \right)$.

Proof is omitted because it is almost identical to that of the proposition 3 in Bao and Kan (2013, pp. 237–243), but by using (A25) in place of (A23).

### Remark

Conditions for the existence of the above moment were stated by Smith (1989) for positive definite $\textbf{B}, \textbf{D}$; in the notation here, the moment exists if and only if $\frac{p}{2} + l > m + n$. For positive semidefinite $\textbf{B}, \textbf{D}$, dealing with their null spaces is critical (see Roberts 1995). Deriving general existence conditions in that case does not seem straightforward, so only the following simple cases are noted here. *(i) When* $N \left( \textbf{D} \right) \subseteq N \left( \textbf{B} \right)$ (or $N \left( \textbf{B} \right) \subseteq N \left( \textbf{D} \right)$): In the former case, it is possible to find a positive constant $\epsilon$ that always satisfies $(\textbf{x}^T \textbf{B} \textbf{x})^m (\textbf{x}^T \textbf{D} \textbf{x})^n \ge (\epsilon \textbf{x}^T \textbf{B} \textbf{x})^{m + n}$, so that $(\textbf{x}^T \textbf{A} \textbf{x})^l / \left[ (\textbf{x}^T \textbf{B} \textbf{x})^m (\textbf{x}^T \textbf{D} \textbf{x})^n \right] \le \epsilon ^{-(m + n)} (\textbf{x}^T \textbf{A} \textbf{x})^l / (\textbf{x}^T \textbf{B} \textbf{x})^{m + n}$. The desired moment of the left-hand side exists if and only if that of the right-hand side does, and the necessary and sufficient condition for the latter is given in the proposition 1 of Bao and Kan (2013), which encompasses the positive definite case above. The same argument applies when $N \left( \textbf{B} \right) \subseteq N \left( \textbf{D} \right)$ with obvious modifications. *(ii) When* $N \left( \textbf{D} \right) \nsubseteq N \left( \textbf{B} \right)$ *and* $N \left( \textbf{B} \right) \nsubseteq N \left( \textbf{D} \right)$: Consider the space $F = \lbrace \textbf{y}: \textbf{y} \perp N \left( \textbf{B} \right) , \textbf{y} \perp N \left( \textbf{D} \right) , \textbf{y} \in {\mathbb {R}}^p \rbrace$. Assuming $F \ne \varnothing$, it is possible to find a positive semi-definite matrix $\textbf{F}$ that always satisfies $(\textbf{x}^T \textbf{A} \textbf{x})^l / \left[ (\textbf{x}^T \textbf{B} \textbf{x})^m (\textbf{x}^T \textbf{D} \textbf{x})^n \right] \le (\textbf{x}^T \textbf{A} \textbf{x})^l / (\textbf{x}^T \textbf{F} \textbf{x})^{m + n}$, by choosing arbitrary small positive numbers and a basis of *F* as its nonzero eigenvalues and eigenvectors, respectively. The proposition 1 of Bao and Kan (2013) applied to the right-hand side defines a sufficient condition for the existence of the moment. This is admittedly a restrictive condition, and the existence condition in the case $F = \varnothing$ is not always obvious.

In the present applications, the moment existence conditions can be easily confirmed for ${\bar{e}}$, ${\bar{c}}$, ${\bar{r}}$, and ${\bar{d}}$ by directly applying the proposition 1 of Bao and Kan (2013). ${\bar{a}}$ and ${\bar{f}}$ always fall within the case (i) above, and the moments exist as long as they are defined (in case of ${\bar{f}}$, $\varvec{\upbeta }$ should not be restricted onto $N \left( \textbf{G} \right)$). On the other hand, ${\bar{\rho }}$ may fall within either (i) or (ii) depending on the structures of the two $\textbf{G}$’s. Even in the latter case and if $F = \varnothing$, which is a pathologic condition in this application, this specific moment exists as long as $\rho$ is defined with probability 1, since $-1 \le \rho \le 1$ whenever defined (above). In addition, the same criteria can be used to confirm that these evolvability measures have finite second moments, which formally validate the use of Monte Carlo evaluations.

## Appendix C Evolvability measures in general normal cases

### C.1 Conditions considered

This section considers a general *p*-variate normality condition for the selection gradient, $\varvec{\upbeta } \sim N_p \left( \varvec{\upeta }, \varvec{\Sigma } \right)$. Regarding ratios of quadratic forms, however, tractable results are available only for normal variables with a spherical covariance structure, i.e., $N_q \left( \varvec{\upmu }, \textbf{I}_q \right)$ for some *q* and arbitrary $\varvec{\upmu }$. Let $\textbf{A}$ be an arbitrary $p \times p$ symmetric matrix and $\textbf{K}$ be a $p \times q$ matrix that satisfies $\textbf{K} \textbf{K}^T = \varvec{\Sigma }$, where *q* ($\le p$) is the rank of $\varvec{\Sigma }$. Writing $\varvec{\upbeta } = \varvec{\upeta } + \textbf{K} \varvec{\upbeta }_0$ with $\varvec{\upbeta }_0 \sim N_q (\textbf{0}_q, \textbf{I}_q)$, the quadratic form in $\varvec{\upbeta }$ with respect to $\textbf{A}$ can be written as $\varvec{\upbeta }^T \textbf{A} \varvec{\upbeta } = (\varvec{\upeta } + \textbf{K} \varvec{\upbeta }_0)^T \textbf{A} (\varvec{\upeta } + \textbf{K} \varvec{\upbeta }_0)$ (see also Mathai and Provost 1992, chapter 3). Certain assumptions are required to express this quadratic form as one in normal variables with a spherical covariance structure.

*(1) When* $\varvec{\Sigma }$ *is nonsingular* In this case, $p = q$ and $\textbf{K}$ is invertible. It suffices to consider the transformation $\varvec{\upbeta }_* = \textbf{K}^{-1} \varvec{\upbeta } \sim N_p \left( \varvec{\upmu }, \textbf{I}_p \right)$, where $\varvec{\upmu } = \textbf{K}^{-1} \varvec{\upeta }$. The quadratic form can be rewritten as $\varvec{\upbeta }^T \textbf{A} \varvec{\upbeta } = \varvec{\upbeta }_*^T \textbf{K}^T \textbf{A} \textbf{K} \varvec{\upbeta }_*$, a new quadratic form in $\varvec{\upbeta }_*$ with respect to $\textbf{K}^T \textbf{A} \textbf{K}$, to which results below apply.

*(2) When* $\varvec{\Sigma }$ *is singular* In this case, $\textbf{K}$ is not invertible and at least one of the following additional conditions need to be satisfied for the results below to be applicable. *(i)* $\varvec{\upeta } \in R \left( \varvec{\Sigma } \right)$ (*including* $\varvec{\upeta } = \textbf{0}_p$): Under this condition, it holds that $\varvec{\upeta } = \textbf{K} \textbf{K}^- \varvec{\upeta }$ (e.g., Schott 2016, theorem 5.25) since $R \left( \varvec{\Sigma } \right) = R \left( \textbf{K} \right)$. It is then possible to consider $\varvec{\upbeta }^T \textbf{A} \varvec{\upbeta } = \varvec{\upbeta }_*^T \textbf{K}^T \textbf{A} \textbf{K} \varvec{\upbeta }_*$ with $\varvec{\upbeta }_* = \textbf{K}^- \varvec{\upbeta } \sim N_p \left( \varvec{\upmu }, \textbf{I}_p \right)$, where $\varvec{\upmu } = \textbf{K}^- \varvec{\upeta }$, as a generalization of the nonsingular case. *(ii)* $R \left( \textbf{A} \right) \subseteq R \left( \varvec{\Sigma } \right)$: From the symmetry of $\textbf{A}$ and the fact that $R \left( \textbf{K} \right) = R \left( {\textbf{K}^-}^T \right)$, it holds that $\textbf{A} = {\textbf{K}^-}^T \textbf{K}^T \textbf{A} \textbf{K} \textbf{K}^-$ under this condition (Schott 2016, theorem 5.25). Then the same transformation as for *(i)* applies. (Utility of this condition in the present applications is relatively limited, since all evolvability measures except $\rho$ involve at least one $\textbf{I}_p$ in place of $\textbf{A}$.) *(iii)* $\textbf{A} \varvec{\upeta } = \textbf{0}_p$: Under this condition, the above quadratic form simplifies into $\varvec{\upbeta }_0^T \textbf{K}^T \textbf{A} \textbf{K} \varvec{\upbeta }_0$, to which results below apply with $\varvec{\upmu } = \textbf{0}_q$.

In practice, the conditions (1) or (2)(i) above seem to cover most cases of biological interest. That is, if the variation of selection gradient $\varvec{\upbeta }$ is restricted to a certain subspace (condition (2)), then it seems plausible that the mean selection gradient $\varvec{\upeta }$ is also in the same subspace (condition (2)(i)). Otherwise, $\varvec{\upbeta }$ ought to contain a nonzero deterministic component ($\varvec{\upeta }$) that is strictly unaffected by (linearly independent of) its potential variability (represented by $\varvec{\Sigma }$).

### C.2 Average evolvability measures

Expressions for average evolvability measures for $\varvec{\upbeta } \sim N_p \left( \varvec{\upeta }, \varvec{\Sigma } \right)$ or equivalently $\varvec{\upbeta }_* \sim N_q \left( \varvec{\upmu }, \textbf{I}_q \right)$ can be derived from Propositions 1 and 4.

The average evolvability ${\bar{e}}$ is

C29 $$\begin{aligned} {\bar{e}}&= {{\,\textrm{E}\,}}_{\varvec{\upbeta }}\! \left( \frac{ \varvec{\upbeta }^T \textbf{G} \varvec{\upbeta } }{ \varvec{\upbeta }^T \varvec{\upbeta } } \right) = {{\,\textrm{E}\,}}_{\varvec{\upbeta }_*}\! \left( \frac{ \varvec{\upbeta }_*^T \textbf{K}^T \textbf{G} \textbf{K} \varvec{\upbeta }_* }{ \varvec{\upbeta }_*^T \textbf{K}^T \textbf{K} \varvec{\upbeta }_* } \right) \nonumber \\&= \alpha e^{ -\frac{\varvec{\upmu }^T\varvec{\upmu }}{2} } \sum _{j=0}^\infty \sum _{k=0}^\infty \frac{ \left( \frac{1}{2} \right) _{j + k + 1} }{ 2^k \left( \frac{1}{2} \right) _{k} \left( \frac{q}{2} + k \right) _{j + 1} k! } C^{\left[ {1} \right] , \left[ {j} \right] , \left[ {k} \right] }_{\left[ {j + k + 1} \right] } \! \left( \textbf{K}^T \textbf{G} \textbf{K} , \textbf{I}_q - \alpha \textbf{K}^T \textbf{K} , \varvec{\upmu } \varvec{\upmu }^T \right) \nonumber \\&= \alpha \sum _{j=0}^\infty \frac{\left( 1 \right) _{j}}{\left( \frac{q}{2} \right) _{j+1}} {\tilde{h}}_{1; j} \! \left( \textbf{K}^T \textbf{G} \textbf{K} ; \textbf{I}_q - \alpha \textbf{K}^T \textbf{K} \right) , \end{aligned}$$

where $\alpha$ is a positive constant that satisfies $0< \alpha < 2 / \lambda _{\max } \! \left( \textbf{K}^T \textbf{K} \right)$.

As discussed in the text (2.4), the average conditional evolvability ${\bar{c}}$ and average autonomy ${\bar{a}}$ can be nonzero only if the distribution of $\varvec{\upbeta }$ is strictly within $R \left( \textbf{G} \right)$. This happens (i) when $\textbf{G}$ is nonsingular; or (ii) when $\textbf{G}$ is singular, $\varvec{\upeta } \in R \left( \textbf{G} \right)$ and $R \left( \varvec{\Sigma } \right) \subseteq R \left( \textbf{G} \right)$.

Under these conditions, the following expression can be obtained for the average conditional evolvability ${\bar{c}}$:

C30 $$\begin{aligned} {\bar{c}}&= {{\,\textrm{E}\,}}_{\varvec{\upbeta }}\! \left( \frac{\varvec{\upbeta }^T \varvec{\upbeta }}{\varvec{\upbeta }^T \textbf{G}^- \varvec{\upbeta }} \right) = {{\,\textrm{E}\,}}_{\varvec{\upbeta }_*}\! \left( \frac{ \varvec{\upbeta }_*^T \textbf{K}^T \textbf{K} \varvec{\upbeta }_* }{ \varvec{\upbeta }_*^T \textbf{K}^T \textbf{G}^{-} \textbf{K} \varvec{\upbeta }_* } \right) \nonumber \\&= \alpha e^{ - \frac{\varvec{\upmu }^T\varvec{\upmu }}{2} } \sum _{j=0}^{\infty } \sum _{k=0}^{\infty }\frac{ \left( \frac{1}{2} \right) _{j + k + 1} }{ 2^k \left( \frac{1}{2} \right) _{k} \left( \frac{q}{2} + k \right) _{j + 1} k! } \nonumber \\&\quad \cdot C^{\left[ {1} \right] , \left[ {j} \right] , \left[ {k} \right] }_{\left[ {j + k + 1} \right] } \! \left( \textbf{K}^T \textbf{K} , \textbf{I}_q - \alpha \textbf{K}^T \textbf{G}^{-} \textbf{K} , \varvec{\upmu } \varvec{\upmu }^T \right) \nonumber \\&= \alpha \sum _{j=0}^{\infty } \frac{ \left( 1 \right) _{j} }{ \left( \frac{q}{2} \right) _{j+1} } {\tilde{h}}_{1; j} \! \left( \textbf{K}^T \textbf{K} ; \textbf{I}_q - \alpha \textbf{K}^T \textbf{G}^{-} \textbf{K} \right) , \end{aligned}$$

where $\alpha$ is to satisfy $0< \alpha < 2 / \lambda _{\max } \! \left( \textbf{K}^T \textbf{G}^{-} \textbf{K} \right)$.

Under the same conditions, the average autonomy ${\bar{a}}$ is

C31 $$\begin{aligned} {\bar{a}}&= {{\,\textrm{E}\,}}_{\varvec{\upbeta }}\! \left( \frac{(\varvec{\upbeta }^T \varvec{\upbeta })^2}{\varvec{\upbeta }^T \textbf{G} \varvec{\upbeta } \varvec{\upbeta }^T \textbf{G}^- \varvec{\upbeta }} \right) = {{\,\textrm{E}\,}}_{\varvec{\upbeta }_*}\! \left( \frac{(\varvec{\upbeta }_*^T \textbf{K}^T \textbf{K} \varvec{\upbeta }_*)^2}{\varvec{\upbeta }_*^T \textbf{K}^T \textbf{G} \textbf{K} \varvec{\upbeta }_* \varvec{\upbeta }_*^T \textbf{K}^T \textbf{G}^{-} \textbf{K} \varvec{\upbeta }_*} \right) \nonumber \\&= \alpha _1 \alpha _2 e^{ - \frac{\varvec{\upmu }^T\varvec{\upmu }}{2} } \sum _{i=0}^{\infty } \sum _{j=0}^{\infty } \sum _{k=0}^{\infty } \frac{ \left( \frac{1}{2} \right) _{i + j + k + 2} }{ 2^k \left( \frac{1}{2} \right) _{k} \left( \frac{q}{2} + k \right) _{i + j + 2} k! } \nonumber \\&\quad \cdot C^{\left[ {2} \right] , \left[ {i} \right] , \left[ {j} \right] , \left[ {k} \right] }_{\left[ {i + j + k + 2} \right] } \! \left( \textbf{K}^T \textbf{K} , \textbf{I}_q - \alpha _1 \textbf{K}^T \textbf{G} \textbf{K} , \textbf{I}_q - \alpha _2 \textbf{K}^T \textbf{G}^{-} \textbf{K} , \varvec{\upmu } \varvec{\upmu }^T \right) \nonumber \\&= 2 \alpha _1 \alpha _2 \sum _{i=0}^{\infty } \sum _{j=0}^{\infty } \frac{ \left( 1 \right) _{i} \left( 1 \right) _{j} }{ \left( \frac{q}{2} \right) _{i + j + 2} } {\tilde{h}}_{2; i, j} \! \left( \textbf{K}^T \textbf{K} ; \textbf{I}_q - \alpha _1 \textbf{K}^T \textbf{G} \textbf{K} , \textbf{I}_q - \alpha _2 \textbf{K}^T \textbf{G}^{-} \textbf{K} \right) , \end{aligned}$$

where $\alpha _1$ and $\alpha _2$ are to satisfy $0< \alpha _1 < 2 / \lambda _{\max } \! \left( \textbf{K}^T \textbf{G} \textbf{K} \right)$ and $0< \alpha _2 < 2 / \lambda _{\max } \! \left( \textbf{K}^T \textbf{G}^{-} \textbf{K} \right)$. As before, the average integration ${\bar{i}}$ is $1 - {\bar{a}}$.

The average respondability ${\bar{r}}$ is

C32 $$\begin{aligned} {\bar{r}}= & {} {{\,\textrm{E}\,}}_{\varvec{\upbeta }}\! \left( \sqrt{ \frac{\varvec{\upbeta }^T \textbf{G}^2 \varvec{\upbeta }}{\varvec{\upbeta }^T \varvec{\upbeta }} } \right) = {{\,\textrm{E}\,}}_{\varvec{\upbeta }_*}\! \left( \sqrt{ \frac{\varvec{\upbeta }_*^T \textbf{K}^T \textbf{G}^2 \textbf{K}^T \varvec{\upbeta }_*}{\varvec{\upbeta }_*^T \textbf{K}^T \textbf{K} \varvec{\upbeta }_*} } \right) \nonumber \\= & {} \alpha _1^{-\frac{1}{2}} \alpha _2^{\frac{1}{2}} e^{ - \frac{\varvec{\upmu }^T\varvec{\upmu }}{2} } \sum _{i=0}^{\infty } \sum _{j=0}^{\infty } \sum _{k=0}^{\infty } \frac{ \left( -\frac{1}{2} \right) _{i} \left( \frac{1}{2} \right) _{j} \left( \frac{1}{2} \right) _{i+j+k} }{ 2^k \left( \frac{1}{2} \right) _{k} \left( \frac{q}{2} + k \right) _{i + j} i! j! k! } \nonumber \\{} & {} \quad \cdot C^{\left[ {i} \right] , \left[ {j} \right] , \left[ {k} \right] }_{\left[ {i + j + k} \right] } \! \left( \textbf{I}_q - \alpha _1 \textbf{K}^T \textbf{G}^2 \textbf{K} , \textbf{I}_q - \alpha _2 \textbf{K}^T \textbf{K} , \varvec{\upmu } \varvec{\upmu }^T \right) \nonumber \\= & {} \alpha _1^{-\frac{1}{2}} \alpha _2^{\frac{1}{2}} \sum _{i=0}^{\infty } \sum _{j=0}^{\infty } \frac{ \left( -\frac{1}{2} \right) _{i} \left( \frac{1}{2} \right) _{j} }{ \left( \frac{q}{2} \right) _{i + j} } h_{i, j} \! \left( \textbf{I}_q - \alpha _1 \textbf{K}^T \textbf{G}^2 \textbf{K} , \textbf{I}_q - \alpha _2 \textbf{K}^T \textbf{K} \right) , \end{aligned}$$

where $\alpha _1$ and $\alpha _2$ are to satisfy $0< \alpha _1 < 2 / \lambda _{\max } \! \left( \textbf{K}^T \textbf{G}^2 \textbf{K} \right)$ and $0< \alpha _2 < 2 / \lambda _{\max } \! \left( \textbf{K}^T\textbf{K} \right)$.

The average flexibility ${\bar{f}}$ is

C33 $$\begin{aligned} {\bar{f}}&= {{\,\textrm{E}\,}}_{\varvec{\upbeta }}\! \left( \frac{ \varvec{\upbeta }^T \textbf{G} \varvec{\upbeta } }{ \sqrt{ \varvec{\upbeta }^T \varvec{\upbeta } \varvec{\upbeta }^T \textbf{G}^2 \varvec{\upbeta } } } \right) = {{\,\textrm{E}\,}}_{\varvec{\upbeta }_*}\! \left( \frac{ \varvec{\upbeta }_*^T \textbf{K}^T \textbf{G} \textbf{K} \varvec{\upbeta }_* }{ \sqrt{ \varvec{\upbeta }_*^T \textbf{K}^T \textbf{K} \varvec{\upbeta }_* \varvec{\upbeta }_*^T \textbf{K}^T \textbf{G}^2 \textbf{K} \varvec{\upbeta }_* } } \right) \nonumber \\&= (\alpha _1 \alpha _2)^{\frac{1}{2}} e^{ - \frac{\varvec{\upmu }^T\varvec{\upmu }}{2} } \sum _{i=0}^{\infty } \sum _{j=0}^{\infty } \sum _{k=0}^{\infty } \frac{ \left( \frac{1}{2} \right) _{i} \left( \frac{1}{2} \right) _{j} \left( \frac{1}{2} \right) _{i + j + k + 1} }{ 2^k \left( \frac{1}{2} \right) _{k} \left( \frac{q}{2} + k \right) _{i + j + 1} i! j! k! } \nonumber \\&\quad \cdot C^{\left[ {1} \right] , \left[ {i} \right] , \left[ {j} \right] , \left[ {k} \right] }_{\left[ {i + j + k + 1} \right] } \! \left( \textbf{K}^T \textbf{G} \textbf{K} , \textbf{I}_q - \alpha _1 \textbf{K}^T \textbf{K} , \textbf{I}_q - \alpha _2 \textbf{K}^T \textbf{G}^2 \textbf{K} , \varvec{\upmu } \varvec{\upmu }^T \right) \nonumber \\&= (\alpha _1 \alpha _2)^{\frac{1}{2}} \sum _{i=0}^{\infty } \sum _{j=0}^{\infty } \frac{ \left( \frac{1}{2} \right) _{i} \left( \frac{1}{2} \right) _{j} }{ \left( \frac{q}{2} \right) _{i + j + 1} } {\tilde{h}}_{1; i, j} \! \left( \textbf{K}^T \textbf{G} \textbf{K} ; \textbf{I}_q - \alpha _1 \textbf{K}^T \textbf{K} , \textbf{I}_q - \alpha _2 \textbf{K}^T \textbf{G}^2 \textbf{K} \right) , \end{aligned}$$

where $\alpha _1$ and $\alpha _2$ are to satisfy $0< \alpha _1 < 2 / \lambda _{\max } \! \left( \textbf{K}^T \textbf{K} \right)$ and $0< \alpha _2 < 2 / \lambda _{\max } \! \left( \textbf{K}^T \textbf{G}^2 \textbf{K} \right)$.

The average response difference ${\bar{d}}$ is

C34 $$\begin{aligned} {\bar{d}}&= {{\,\textrm{E}\,}}_{\varvec{\upbeta }}\! \left( \sqrt{ \frac{\varvec{\upbeta }^T \left( \textbf{G}_1 - \textbf{G}_2 \right) ^2 \varvec{\upbeta }}{\varvec{\upbeta }^T \varvec{\upbeta }} } \right) = {{\,\textrm{E}\,}}_{\varvec{\upbeta }_*}\! \left( \sqrt{ \frac{\varvec{\upbeta }_*^T \textbf{K}^T \left( \textbf{G}_1 - \textbf{G}_2 \right) ^2 \textbf{K} \varvec{\upbeta }_*}{\varvec{\upbeta }_*^T \textbf{K}^T \textbf{K} \varvec{\upbeta }_*} } \right) \nonumber \\&= \alpha _1^{-\frac{1}{2}} \alpha _2^{\frac{1}{2}} e^{ - \frac{\varvec{\upmu }^T\varvec{\upmu }}{2} } \sum _{i=0}^{\infty } \sum _{j=0}^{\infty } \sum _{k=0}^{\infty } \frac{ \left( -\frac{1}{2} \right) _{i} \left( \frac{1}{2} \right) _{j} \left( \frac{1}{2} \right) _{i + j + k} }{ 2^k \left( \frac{1}{2} \right) _{k} \left( \frac{q}{2} + k \right) _{i + j} i! j! k! } \nonumber \\&\quad \cdot C^{\left[ {i} \right] , \left[ {j} \right] , \left[ {k} \right] }_{\left[ {i + j + k} \right] } \! \left( \textbf{I}_q - \alpha _1 \textbf{K}^T \left( \textbf{G}_1 - \textbf{G}_2 \right) ^2 \textbf{K} , \textbf{I}_q - \alpha _2 \textbf{K}^T \textbf{K} , \varvec{\upmu } \varvec{\upmu }^T \right) \nonumber \\&= \alpha _1^{-\frac{1}{2}} \alpha _2^{\frac{1}{2}} \sum _{i=0}^{\infty } \sum _{j=0}^{\infty } \frac{ \left( -\frac{1}{2} \right) _{i} \left( \frac{1}{2} \right) _{j} }{ \left( \frac{q}{2} \right) _{i + j} } h_{i, j} \! \left( \textbf{I}_q - \alpha _1 \textbf{K}^T \left( \textbf{G}_1 - \textbf{G}_2 \right) ^2 \textbf{K} , \textbf{I}_q - \alpha _2 \textbf{K}^T \textbf{K} \right) , \end{aligned}$$

where $\alpha _1$ and $\alpha _2$ are to satisfy $0< \alpha _1 < 2 / \lambda _{\max } \! \left( \textbf{K}^T \left( \textbf{G}_1 - \textbf{G}_2 \right) ^2 \textbf{K} \right)$ and $0< \alpha _2 < 2 / \lambda _{\max } \! \left( \textbf{K}^T\textbf{K} \right)$.

The average response correlation ${\bar{\rho }}$ is

C35 $$\begin{aligned} {\bar{\rho }}&= {{\,\textrm{E}\,}}_{\varvec{\upbeta }}\! \left( \frac{ \varvec{\upbeta }^T \textbf{G}_1 \textbf{G}_2 \varvec{\upbeta } }{ \sqrt{ \varvec{\upbeta }^T \textbf{G}_1^2 \varvec{\upbeta } \varvec{\upbeta }^T \textbf{G}_2^2 \varvec{\upbeta } } } \right) = {{\,\textrm{E}\,}}_{\varvec{\upbeta }_*}\! \left( \frac{ \varvec{\upbeta }_*^T \textbf{K}^T \textbf{G}_1 \textbf{G}_2 \textbf{K} \varvec{\upbeta }_* }{ \sqrt{ \varvec{\upbeta }_*^T \textbf{K}^T \textbf{G}_1^2 \textbf{K} \varvec{\upbeta }_* \varvec{\upbeta }_*^T \textbf{K}^T \textbf{G}_2^2 \textbf{K} \varvec{\upbeta }_* } } \right) \nonumber \\&= (\alpha _1 \alpha _2)^{\frac{1}{2}} e^{ - \frac{\varvec{\upmu }^T\varvec{\upmu }}{2} } \sum _{i=0}^{\infty } \sum _{j=0}^{\infty } \sum _{k=0}^{\infty } \frac{ \left( \frac{1}{2} \right) _{i} \left( \frac{1}{2} \right) _{j} \left( \frac{1}{2} \right) _{i + j + k + 1} }{ 2^k \left( \frac{1}{2} \right) _{k} \left( \frac{q}{2} + k \right) _{i + j + 1} i! j! k! } \nonumber \\&\quad \cdot C^{\left[ {1} \right] , \left[ {i} \right] , \left[ {j} \right] , \left[ {k} \right] }_{\left[ {i + j + k + 1} \right] } \! \left( \textbf{K}^T \textbf{G}_{12} \textbf{K} , \textbf{I}_q - \alpha _1 \textbf{K}^T \textbf{G}_1^2 \textbf{K} , \textbf{I}_q - \alpha _2 \textbf{K}^T \textbf{G}_2^2 \textbf{K} , \varvec{\upmu } \varvec{\upmu }^T \right) \nonumber \\&= (\alpha _1 \alpha _2)^{\frac{1}{2}} \sum _{i=0}^{\infty } \sum _{j=0}^{\infty } \frac{ \left( \frac{1}{2} \right) _{i} \left( \frac{1}{2} \right) _{j} }{ \left( \frac{q}{2} \right) _{i + j + 1} } \nonumber \\&\quad \cdot {\tilde{h}}_{1; i, j} \! \left( \textbf{K}^T \textbf{G}_{12} \textbf{K} ; \textbf{I}_q - \alpha _1 \textbf{K}^T \textbf{G}_1^2 \textbf{K} , \textbf{I}_q - \alpha _2 \textbf{K}^T \textbf{G}_2^2 \textbf{K} \right) \end{aligned}$$

where $\textbf{G}_{12} = \left( \textbf{G}_1 \textbf{G}_2 + \textbf{G}_2 \textbf{G}_1 \right) / 2$, and $\alpha _1$ and $\alpha _2$ are to satisfy $0< \alpha _1 < 2 / \lambda _{\max } \! \left( \textbf{K}^T \textbf{G}_1^2 \textbf{K} \right)$ and $0< \alpha _2 < 2 / \lambda _{\max } \! \left( \textbf{K}^T \textbf{G}_2^2 \textbf{K} \right)$.

### C.3 Practical evaluation and truncation error

The above series expressions for average measures in general cases can be evaluated by one of the recursive algorithms described by Hillier et al. (2009, 2014) and Bao and Kan (2013). When the mean $\varvec{\upmu }$ is nonzero, the series can be most efficiently evaluated by recursions for $h_{i,j}$ and ${\tilde{h}}_{i,j}$ described in Hillier et al. (2014, section 5.4) and Bao and Kan (2013, section 5). When possible, it is recommended to inspect a profile of the partial sum across varying orders as the signs of the terms can in general fluctuate in those series.

At present, truncation error bounds for general cases are available only for ${\bar{e}}$ and ${\bar{c}}$. From the theorem 7 of Hillier et al. (2014), a truncation error bound for these average measures evaluated up to $k = M$ is obtained from the following, by inserting $(\textbf{A}, \textbf{B}) = (\textbf{K}^T \textbf{G} \textbf{K}, \textbf{K}^T \textbf{K})$ and $(\textbf{K}^T \textbf{K}, \textbf{K}^T \textbf{G}^- \textbf{K})$ for ${\bar{e}}$ and ${\bar{c}}$, respectively:

C36 $$\begin{aligned} \alpha ^n \frac{\left( n \right) _{M+1}}{\left( \frac{q}{2} \right) _{M+2}} \left( \left|\alpha \textbf{B} \right|^{-\frac{1}{2}} \left[ \frac{{{\,\textrm{tr}\,}}\left( \textbf{B}^{-1} \textbf{A} \right) + t \alpha ^{-1} \varvec{\upmu }^T \textbf{B}^{-1} \textbf{A} \textbf{B}^{-1} \varvec{\upmu } }{2} \right] \right. \nonumber \\ \left. \cdot \exp {\left( \frac{ t \alpha ^{-1} \varvec{\upmu }^T \textbf{B}^{-1} \varvec{\upmu } - \varvec{\upmu }^T \varvec{\upmu } }{2} \right) } - S_M \right) . \end{aligned}$$

Here, $n = 1$, $t = 2$, and $S_M = \sum _{j=0}^M {\hat{h}}_{1; j} \! \left( \textbf{A} ; \textbf{I}_q - \alpha \textbf{B} \right)$, with ${\hat{h}}_{1; j} \! \left( \textbf{A}_1; \textbf{A}_2 \right)$ being the coefficient of $t_1 t_2^j$ in the expansion

$$\begin{aligned}{} & {} \left| \textbf{I}_q - t_1 \textbf{A}_1 - t_2 \textbf{A}_2 \right| \exp { \left( \frac{ (1 + t_2) \varvec{\upmu }^T \left( \textbf{I}_q - t_1 \textbf{A}_1 - t_2 \textbf{A}_2 \right) ^{-1} \varvec{\upmu } - \varvec{\upmu }^T \varvec{\upmu } }{2} \right) } \\{} & {} \quad = \sum _{i=0}^\infty \sum _{j=0}^\infty {\hat{h}}_{i; j} \! \left( \textbf{A}_1; \textbf{A}_2 \right) t_1^i t_2^j, \end{aligned}$$

which can be evaluated by a recursive algorithm similar to the one presented in Hillier et al. (2014, theorem 6).^6^ This expression bounds the truncation error in absolute values unlike those in (19)–(22) which are one-sided. This is because ${\tilde{h}}_{i,j}$ can take both positive and negative values when $\varvec{\upmu }$ is nonzero, so that the partial sums are not always increasing.

Under the restriction $\textbf{K}^T \textbf{K} = \textbf{I}_q$, a similar error bound can be derived for ${\bar{f}}$, by letting $\textbf{A} = \textbf{K}^T \textbf{G} \textbf{K}$, $\textbf{B} = \textbf{K}^T \textbf{G}^2 \textbf{K}$, $n = 1/2$, $t = 3$, and $S_M = \sum _{k=0}^{M} \sum _{l=0}^{k} {\hat{h}}_{1; l, k - l} \! \left( \textbf{A} ; \textbf{I}_q - \alpha \textbf{B} , \textbf{0}_{q \times q} \right)$ in (C36). Here, ${\hat{h}}_{1; j, k} \! \left( \textbf{A}_1; \textbf{A}_2, \textbf{A}_3 \right)$ is the coefficient of $t_1 t_2^j t_3^{k}$ in the expansion of

$$\begin{aligned}{} & {} \left| \textbf{I}_q - t_1 \textbf{A}_1 - t_2 \textbf{A}_2 - t_3 \textbf{A}_3 \right|\\{} & {} \cdot \exp { \left( \frac{ (1 + t_2 + t_3) \varvec{\upmu }^T \left( \textbf{I}_q - t_1 \textbf{A}_1 - t_2 \textbf{A}_2 - t_3 \textbf{A}_3 \right) ^{-1} \varvec{\upmu } - \varvec{\upmu }^T \varvec{\upmu } }{2} \right) }\\{} & {} =\sum _{i=0}^\infty \sum _{j=0}^\infty \sum _{k=0}^\infty {\hat{h}}_{i; j, k} \! \left( \textbf{A}_1; \textbf{A}_2, \textbf{A}_3 \right) t_1^i t_2^j t_3^{k}, \end{aligned}$$

which can be evaluated by the same recursive algorithm for ${\hat{h}}_{i; j}$ with obvious modifications.

## Appendix D Connections to previous results

### D.1 Average conditional evolvability in two traits

Hansen and Houle (2008) stated that the average conditional evolvability, under the uniform distribution of $\textbf{u}$ on $S^{p - 1}$, equals to the geometric mean of the eigenvalues of $\textbf{G}$ when $p = 2$. It is possible to confirm that the present result is consistent with their argument as follows. From (13),

$$\begin{aligned} {\bar{c}}&= \alpha \, {}_2F_1 \left( \frac{1}{2}, 1; \frac{2}{2} ; \textbf{I}_p - \alpha \textbf{G}^{-1} \right) \\&= \alpha \, {}_1F_0 \left( \frac{1}{2}; \textbf{I}_p - \alpha \textbf{G}^{-1} \right) \\&= \alpha \left| \textbf{I}_p - \left( \textbf{I}_p - \alpha \textbf{G}^{-1} \right) \right|^{-\frac{1}{2}} \\&= \left| \textbf{G} \right|^{\frac{1}{2}}, \end{aligned}$$

where in the second equation the denominator parameter cancels with one of the numerator parameters, and the third equation is from (A10). Since the determinant is equal to the product of the eigenvalues, this proves the argument.

### D.2 Average respondability under complete integration

Kirkpatrick (2009) considered the average selection response ${\bar{R}}$, which equals ${\bar{r}} / \lambda _{\max } \! \left( \textbf{G} \right)$ here when $\textbf{u}$ is uniformly distributed on $S^{p - 1}$. This section shows that the present expression for ${\bar{r}}$ (15) entails his result for ${\bar{R}}$ under the case of complete integration, $\textbf{G} = {{\,\textrm{diag}\,}}\left( \lambda _{\max }, 0, \dots , 0 \right)$, the only situation for which an analytic expression was given. The expression is (Kirkpatrick 2009, (6) and (A7) therein)

D37 $$\begin{aligned} {\bar{R}}&= \frac{ 2 \Gamma \left( \frac{p}{2} \right) }{ (p - 1) \sqrt{\pi } \Gamma \left( \frac{p - 1}{2} \right) } \nonumber \\&= \frac{\Gamma \left( \frac{p}{2} \right) }{ \Gamma \left( \frac{1}{2} \right) \Gamma \left( \frac{p + 1}{2} \right) }. \end{aligned}$$

Taking $$\alpha = \lambda _{\max }^{-2}$$, (15) becomes

$$\begin{aligned} {\bar{r}}&= \lambda _{\max } \sum _{k=0}^{\infty } \frac{ \left( -\frac{1}{2} \right) _{k} \left( \frac{1}{2} \right) _{k} }{ \left( \frac{p}{2} \right) _{k} k! } C_{\left[ {k} \right] } \! \left( \textbf{I}_p - {{\,\textrm{diag}\,}}\left( 1, 0, \dots , 0 \right) \right) \\&= \lambda _{\max } \sum _{k=0}^{\infty } \frac{ \left( -\frac{1}{2} \right) _{k} \left( \frac{1}{2} \right) _{k} }{ \left( \frac{p}{2} \right) _{k} k! } \frac{ \left( \frac{p - 1}{2} \right) _{k} }{ \left( \frac{1}{2} \right) _{k} } \\&= \lambda _{\max } \sum _{k=0}^{\infty } \frac{ \left( -\frac{1}{2} \right) _{k} \left( \frac{p - 1}{2} \right) _{k} }{ \left( \frac{p}{2} \right) _{k} k! } \\&= \lambda _{\max } \frac{ \Gamma \left( \frac{p}{2} \right) \Gamma \left( 1 \right) }{ \Gamma \left( \frac{1}{2} \right) \Gamma \left( \frac{p + 1}{2} \right) }, \end{aligned}$$

where the second equation comes from the fact $C_{\left[ {k} \right] } \! \left( {{\,\textrm{diag}\,}}\left( 0, 1, \dots , 1 \right) \right) = C_{\left[ {k} \right] } \! \left( \textbf{I}_{p-1} \right)$ and (A6), and the last equation is from the Gauss hypergeometric theorem: ${}_2F_1 \left( a, b; c; 1 \right) = \frac{\Gamma \left( c \right) \Gamma \left( c - b - a \right) }{\Gamma \left( c - b \right) \Gamma \left( c - a \right) }$. The equivalence to (D37) is obvious.

## Footnotes

1 The term evolvability bears various meanings in the current evolutionary biology (e.g., Brown 2014; Crother and Murray 2019; Feiner et al. 2021; Jablonski 2022; Love et al. 2022; Riederer et al. 2022). In the present paper, the use of this term is restricted to certain mathematical aspects of the $\textbf{G}$ matrix and response vectors—the Hansen–Houle evolvability—unless stated otherwise.

2 A potential alternative to the average measures in the above sense is the average of the evolvability measures calculated from the $\varvec{\upbeta }$’s aligned with all individual traits (Armbruster et al. 2009; Haber 2011; Pavlicev and Hansen 2011; Machado et al. 2018). Although that would be valid for descriptive purposes, its interpretability in the context of multivariate selection might be limited. In any case, the calculation of such averages is straightforward and not concerned herein.

3 Rohlf (2017, p. 544) asserted that $\rho$ cannot be negative because he assumed, inappropriately in this context, the response vectors to have no polarity. Negative $\rho$ can occur when, e.g., $\varvec{\upbeta }$ bisects the wider of the two angles formed by the major axes of highly integrated $\textbf{G}$ matrices. Except for this subtle fallacy, his criticisms on previous interpretations of $\rho$ or ${\bar{\rho }}$ (e.g., Melo et al. 2016) seem largely pertinent (see also Discussion).

4 This confusion apparently arose from the custom of using the same symbol $\varvec{\upbeta }$ for both standardized and unstandardized selection gradients (above). It seems advisable to use a different symbol for the former, e.g., $\textbf{u}$ herein.

## Code availability

R codes to reproduce numerical experiments are provided as the Online Resource 1.

## References

[CR1] Agrawal AF, Stinchcombe JR (2009). How much do genetic covariances alter the rate of adaptation?. Proc R Soc B.

[CR2] Aguirre JD, Hine E, McGuigan K, Blows MW (2014). Comparing **G**: multivariate analysis of genetic variation in multiple populations. Heredity.

[CR3] Armbruster WS, Hansen TF, Pélabon C, Pérez-Barrales R, Maad J (2009). The adaptive accuracy of flowers: measurement and microevolutionary patterns. Ann Bot.

[CR4] Bao Y, Kan R (2013). On the moments of ratios of quadratic forms in normal random variables. J Multivar Anal.

[CR5] Bates D, Eddelbuettel D (2013). Fast and elegant numerical linear algebra using the RcppEigen package. J Stat Softw.

[CR6] Blows MW (2007). A tale of two matrices: multivariate approaches in evolutionary biology. J Evol Biol.

[CR7] Blows M, Walsh B (2009) Spherical cows grazing in flatland: constraints to selection and adaptation. In: van der Werf J, Graser HU, Frankham R, Gondro C (eds) Adaptation and fitness in animal populations: evolutionary and breeding perspectives on genetic resource management. Springer, Dordrecht, pp 83–101. 10.1007/978-1-4020-9005-9_6

[CR8] Bolstad GH, Hansen TF, Pélabon C, Falahati-Anbaran M, Pérez-Baralles R, Armbruster WS (2014). Genetic constraints predict evolutionary divergence in *Dalechampia* blossoms. Philos Trans R Soc B.

[CR9] Brommer JE (2014). Using average autonomy to test whether behavioral syndromes constrain evolution. Behav Ecol Sociobiol.

[CR10] Brown RL (2014). What evolvability really is. British J Philos Sci.

[CR11] Cheverud JM (1982). Phenotypic, genetic, and environmental morphological integration in the cranium. Evolution.

[CR12] Cheverud JM (1989). A comparative analysis of morphological variation patterns in the papionins. Evolution.

[CR13] Cheverud JM (1996). Quantitative genetic analysis of cranial morphology in the cotton-top (*Saguinus oedipus*) and saddle-back (*S. fuscicollis*) tamarins. J Evol Biol.

[CR14] Cheverud JM, Marroig G (2007). Comparing covariance matrices: random skewers method compared to the common principal components model. Genet Mol Biol.

[CR15] Chevin LM (2013). Genetic constraints on adaptation to a changing environment. Evolution.

[CR16] Chikuse Y, Gupta RP (1980). Invariant polynomials with matrix arguments and their applications. Multivariate statistical analysis.

[CR17] Chikuse Y (1987). Methods for constructing top order invariant polynomials. Econom Theor.

[CR18] Chikuse Y (2003). Statistics on special manifolds.

[CR19] Chikuse Y, Davis AW (1986). Some properties of invariant polynomials with matrix arguments and their applications in econometrics. Ann Inst Statist Math.

[CR20] Chikuse Y, Davis AW (1986). A survey on the invariant polynomials with matrix arguments in relation to econometric distribution theory. Econom Theor.

[CR21] Constantine AG (1963). Some non-central distribution problems in multivariate analysis. Ann Math Stat.

[CR22] Costa e Silva J, Potts BM, Harrison PA (2020) Population divergence along a genetic line of least resistance in the tree species *Eucalyptus globulus*. Genes 11:1095. 10.3390/genes11091095 10.3390/genes11091095 PMC7565133 32962131

[CR23] Cressie N, Davis AS, Folks JL, Policello GEII (1981). The moment-generating function and negative integer moments. Am Stat.

[CR24] Crother BI, Murray CM (2019). Early usage and meaning of evolvability. Ecol Evol.

[CR25] Davies PI, Higham NJ (2000). Numerically stable generation of correlation matrices and their factors. BIT Numer Math.

[CR26] Davis AW (1979). Invariant polynomials with two matrix arguments extending the zonal polynomials: applications to multivariate distribution theory. Ann Inst Statist Math.

[CR27] Davis AW, Krishnaiah PR (1980). Invariant polynomials with two matrix arguments, extending the zonal polynomials. Multivariate analysis–V.

[CR28] Delahaie B, Charmantier A, Chantepie S, Garant D, Porlier M, Teplitsky C (2017). Conserved **G**-matrices of morphological and life-history traits among continental and island blue tit populations. Heredity.

[CR29] Feiner N, Jackson ISC, Van der Cruyssen E, Uller T (2021). A highly conserved ontogenetic limb allometry and its evolutionary significance in the adaptive radiation of *Anolis* lizards. Proc R Soc B.

[CR30] Forchini G (2002). The exact cumulative distribution function of a ratio of quadratic forms in normal variables, with application to the AR(1) model. Econom Theor.

[CR31] Forchini G (2005). The distribution of a ratio of quadratic forms in noncentral normal variables. Comm Stat Theory Methods.

[CR32] Grabowski M, Porto A (2017). How many more? Sample size determination in studies of morphological integration and evolvability. Methods Ecol Evol.

[CR33] Gross KI, Richards DSP (1987). Special functions of matrix argument. I: algebraic induction, zonal polynomials, and hypergeometric functions. Trans Am Math Soc.

[CR34] Gupta AK, Kabe DG (1998). Moments of ratios of quadratic forms. Stat Probab Lett.

[CR35] Haber A (2011). A comparative analysis of integration indices. Evol Biol.

[CR36] Haber A (2016). Phenotypic covariation and morphological diversification in the ruminant skull. Am Nat.

[CR37] Hansen TF (2003). Is modularity necessary for evolvability? Remarks on the relationship between pleiotropy and evolvability. Biosystems.

[CR38] Hansen TF, Houle D (2008). Measuring and comparing evolvability and constraint in multivariate characters. J Evol Biol.

[CR39] Hansen TF, Houle D (2009). Corrigendum [of “Measuring and comparing evolvability and constraint in multivariate characters”]. J Evol Biol.

[CR40] Hansen TF, Pélabon C (2021). Evolvability: a quantitative-genetics perspective. Annu Rev Ecol Evol Syst.

[CR41] Hansen TF, Voje KL (2011). Deviation from the line of least resistance does not exclude genetic constraints: a comment on Berner et al. (2010). Evolution.

[CR42] Hansen TF, Armbruster WS, Carlson ML, Pélabon C (2003). Evolvability and genetic constraint in *Dalechampia* blossoms: genetic correlations and conditional evolvability. J Exp Zool B Mol Dev Evol.

[CR43] Hansen TF, Pélabon C, Armbruster WS, Carlson ML (2003). Evolvability and genetic constraint in *Dalechampia* blossoms: components of variance and measures of evolvability. J Evol Biol.

[CR44] Hansen TF, Pélabon C, Houle D (2011). Heritability is not evolvability. Evol Biol.

[CR45] Hansen TF, Solvin TM, Pavlicev M (2019). Predicting evolutionary potential: a numerical test of evolvability measures. Evolution.

[CR46] Hillier G (2001). The density of a quadratic form in a vector uniformly distributed on the $$n$$-sphere. Econom Theor.

[CR47] Hillier G, Kan R, Wang X (2009). Computationally efficient recursions for top-order invariant polynomials with applications. Econom Theor.

[CR48] Hillier G, Kan R, Wang X (2014). Generating functions and short recursions, with applications to the moments of quadratic forms in noncentral normal vectors. Econom Theor.

[CR49] Hine E, Blows MW (2006). Determining the effective dimensionality of the genetic variance-covariance matrix. Genetics.

[CR50] Houle D (1992). Comparing evolvability and variability of quantitative traits. Genetics.

[CR51] Houle D, Meyer K (2015). Estimating sampling error of evolutionary statistics based on genetic covariance matrices using maximum likelihood. J Evol Biol.

[CR52] Hubbe A, Machado FA, Melo D, Garcia G, Sebastião H, Porto A, Cheverud J, Marroig G (2023). Morphological integration during postnatal ontogeny: implications for evolutionary biology. Evolution.

[CR53] Jablonski D (2022). Evolvability and macroevolution: overview and synthesis. Evol Biol.

[CR54] James AT (1960). The distribution of the latent roots of the covariance matrix. Ann Math Stat.

[CR55] James AT (1961). Zonal polynomials of the real positive definite symmetric matrices. Ann Math.

[CR56] James AT (1964). Distributions of matrix variates and latent roots derived from normal samples. Ann Math Stat.

[CR57] Jones MC (1986). Expressions for inverse moments of positive quadratic forms in normal variables. Austral J Stat.

[CR58] Jones MC (1987). On moments of ratios of quadratic forms in normal variables. Stat Probab Lett.

[CR59] Jones AG, Bürger R, Arnold SJ, Hohenlohe PA, Uyeda JC (2012). The effects of stochastic and episodic movement of the optimum on the evolution of the **G**-matrix and the response of the trait mean to selection. J Evol Biol.

[CR60] Kirkpatrick M (2009). Patterns of quantitative genetic variation in multiple dimensions. Genetica.

[CR61] Koev P, Edelman A (2006). The efficient evaluation of the hypergeometric function of a matrix argument. Math Comp.

[CR62] Lande R (1979). Quantitative genetic analysis of multivariate evolution, applied to brain:body size allometry. Evolution.

[CR63] Lande R, Arnold SJ (1983). The measurement of selection on correlated characters. Evolution.

[CR64] Laurent S (2022) HypergeoMat: hypergeometric function of a matrix argument. https://CRAN.R-project.org/package=HypergeoMat

[CR65] Love AC, Grabowski M, Houle D, Liow LH, Porto A, Tsuboi M, Voje KL, Hunt G (2022). Evolvability in the fossil record. Paleobiology.

[CR66] Machado FA (2020). Selection and constraints in the ecomorphological adaptive evolution of the skull of living Canidae (Carnivora, Mammalia). Am Nat.

[CR67] Machado FA, Zahn TMG, Marroig G (2018). Evolution of morphological integration in the skull of Carnivora (Mammalia): changes in Canidae lead to increased evolutionary potential of facial traits. Evolution.

[CR68] Magnus JR (1986). The exact moments of a ratio of quadratic forms in normal variables. Ann Écon Stat.

[CR69] Mardia KV, Jupp PE (1999). Directional statistics.

[CR70] Marroig G, Shirai LT, Porto A, de Oliveira FB, De Conto V (2009). The evolution of modularity in the mammalian skull II: evolutionary consequences. Evol Biol.

[CR71] Mathai AM, Provost SB (1992). Quadratic forms in random variables: theory and applications.

[CR72] Mathai AM, Provost SB, Hayakawa T (1995). Bilinear forms and zonal polynomials.

[CR73] McGlothlin JW, Kobiela ME, Wright HV, Kolbe JJ, Losos JB, Brodie EDIII (2022). Conservation and convergence of genetic architecture in the adaptive radiation of *Anolis* lizards. Am Nat.

[CR74] Melo D, Marroig G (2015). Directional selection can drive the evolution of modularity in complex traits. Proc Natl Acad Sci U S A.

[CR75] Melo D, Garcia G, Hubbe A, Assis AP, Marroig G (2016) EvolQG—an R package for evolutionary quantitative genetics [version 3; referees: 2 approved, 1 approved with reservations]. F1000Research 4:925. 10.12688/f1000research.7082.3 10.12688/f1000research.7082.1 PMC5022708 27785352

[CR76] Meng XL (2005). From unit root to Stein’s estimator to Fisher’s $$k$$ statistics: if you have a moment, I can tell you more. Stat Sci.

[CR77] Meyer K, Kirkpatrick M (2008). Perils of parsimony: properties of reduced-rank estimates of genetic covariance matrices. Genetics.

[CR78] Mezey JG, Houle D (2005). The dimensionality of genetic variation for wing shape in *Drosophila melanogaster*. Evolution.

[CR79] Muirhead RJ (1982). Aspects of multivariate statistical theory.

[CR80] Muirhead RJ, Kotz S, Balakrishnan N, Read C, Vidakovic B, Johnson NL (2006). Zonal polynomials. Encyclopedia of statistical sciences.

[CR81] Opedal ØH, Hildesheim LS, Armbruster WS (2022). Evolvability and constraint in the evolution of three-dimensional flower morphology. Am J Bot.

[CR82] Opedal ØH, Armbruster WS, Hansen TF, Holstad A, Pélabon C, Andersson S, Campbell DR, Caruso CM, Delph LF, Eckert CG (2023). Evolvability and trait function predict phenotypic divergence of plant populations. Proc Natl Acad Sci U S A.

[CR83] Pavlicev M, Hansen TF (2011). Genotype-phenotype maps maximizing evolvability: modularity revisited. Evol Biol.

[CR84] Pavlicev M, Wagner GP, Cheverud JM (2009). Measuring evolutionary constraints through the dimensionality of the phenotype: adjusted bootstrap method to estimate rank of phenotypic covariance matrix. Evol Biol.

[CR85] Pavlicev M, Cheverud JM, Wagner GP (2011). Evolution of adaptive phenotypic variation patterns by direct selection for evolvability. Proc R Soc B.

[CR86] Porto A, de Oliveira FB, Shirai LT, De Conto V, Marroig G (2009). The evolution of modularity in the mammalian skull I: morphological integration patterns and magnitudes. Evol Biol.

[CR87] Puentes A, Granath G, Ågren J (2016). Similarity in **G** matrix structure among natural populations of *Arabidopsis lyrata*. Evolution.

[CR88] R Core Team (2023) R: a language and environment for statistical computing, version 4.2.3. https://www.R-project.org/

[CR89] Revell LJ (2007). The **G** matrix under fluctuating correlational mutation and selection. Evolution.

[CR90] Riederer JM, Tiso S, van Eldijk TJB, Weissing FJ (2022). Capturing the facets of evolvability in a mechanistic framework. Trends Ecol Evol.

[CR91] Roberts LA (1995). On the existence of moments of ratios of quadratic forms. Econom Theor.

[CR92] Rohlf FJ (2017). The method of random skewers. Evol Biol.

[CR93] Rolian C (2009). Integration and evolvability in primate hands and feet. Evol Biol.

[CR94] Saltzberg CJ, Walker LI, Chipps-Walton LE, Costa BMA, Spotorno ÁE, Steppan SJ (2022). Comparative quantitative genetics of the pelvis in four-species of rodents and the conservation of genetic covariance and correlation structure. Evol Biol.

[CR95] Schluter D (1996). Adaptive radiation along genetic lines of least resistance. Evolution.

[CR96] Schott JR (2016). Matrix analysis for statistics.

[CR97] Smith MD (1989). On the expectation of a ratio of quadratic forms in normal variables. J Multivar Anal.

[CR98] Smith MD (1993). Expectations of ratios of quadratic forms in normal variables: evaluating some top-order invariant polynomials. Austral J Stat.

[CR99] Steppan SJ, Phillips PC, Houle D (2002). Comparative quantitative genetics: evolution of the **G** matrix. Trends Ecol Evol.

[CR100] Takemura A (1984). Zonal polynomials, Lecture notes-monograph series.

[CR101] Teplitsky C, Robinson MR, Merilä J (2014) Evolutionary potential and constraints in wild populations. In: Charmantier A, Garant D, Kruuk LEB (eds) Quantitative genetics in the wild. Oxford University Press, Oxford, pp 190–208. 10.1093/acprof:oso/9780199674237.003.0012

[CR102] Walsh B, Blows MW (2009). Abundant genetic variation + strong selection = multivariate genetic constraints: a geometric view of adaptation. Annu Rev Ecol Evol Syst.

[CR103] Watanabe J (2023) qfratio: R package for moments of ratios of quadratic forms using recursion. https://cran.r-project.org/package=qfratio

[CR104] Watanabe J (2022). Statistics of eigenvalue dispersion indices: quantifying the magnitude of phenotypic integration. Evolution.

